# Deciphering the effects of bixin on pulmonary alveolar adenocarcinoma migration and proliferation via targeting BAX/BCL-2 and Cyclin D1

**DOI:** 10.1038/s41598-025-96788-9

**Published:** 2025-04-29

**Authors:** Ressin Varghese, Siva Ramamoorthy

**Affiliations:** School of Bio Sciences and Technology, Vellore Institute of Technology, Vellore, Tamil Nadu 632014 India

**Keywords:** Lung cancer, A549, Bixin, Apoptosis, Cyclin D1, BAX, BCL-2, Cancer, Plant sciences

## Abstract

There is a tremendous upsurge in lung cancer incidences due to changing lifestyles and other environmental risk factors. Unfortunately, the use of clinical therapeutics is causing serious side effects and drug-resistant tumors. Taking account of the severity of lung cancer malignancy and the pressing need for natural therapeutics, we investigated the anticancer potential of bixin in A549, pulmonary alveolar adenocarcinoma cell lines meticulously for the first time. Bixin is an apocarotenoid present in the seed arils of *Bixa orellana* known for its remarkable coloring utilities and high medicinal value. Here, we identified the cytotoxic and anti-migratory nature of bixin through MTT and scratch assay. Bixin also induced characteristic apoptotic morphological changes in cells which were distinguished through 4ʹ,6-diamidino-2-phenylindole (DAPI), and Acridine orange/Ethidium bromide (AO/EB) labeling. Bixin induced the mitochondrion-associated intrinsic apoptosis in A549 cells as evidenced in mitochondrial membrane potential assay, apoptosis assay, cell cycle analysis, and caspase assays. The relative gene expression studies proved that the bixin upregulated BAX, and downregulated BCL-2 and Cyclin D1. The *in-silico* analyses, molecular docking and molecular dynamics simulation underlined the interaction features of bixin and targeted proteins.

## Introduction

Lung cancer is regarded as the most commonly occurring cancer worldwide with approximately 2.5 million new cases accounting for 12.4% of the total new cancer cases in the world. Besides, lung cancer is the most malignant cancer causing 1.8 million deaths, which is 18.7% of the total cancer deaths. Tobacco and alcohol consumption, and obesity are considered the key driving factors of the escalating occurrences of lung cancer, while air pollution is also a major environmental risk factor^1,2^. As per the international standard for histologic classification of lung tumors proposed by the World Health Organization (WHO) and International Association for the Study of Lung Cancer (IASLC), there are four major histologic types of lung cancer i.e., squamous cell carcinoma, adenocarcinoma, small cell carcinoma (SCLC) and large cell carcinoma. Nevertheless, the most important distinction between lung carcinoma is small cell carcinoma and non-small cell lung carcinoma (NSCLC)^3^. NSCLC is a quite widespread form of lung cancer which is evidenced to spread rapidly at an early stage of cancer and known for its insensitiveness to established chemotherapeutic approaches. Surgery is considered the most appropriate strategy to eradicate lung carcinomas. Unfortunately, the concomitant infection risks and cost factor are major problems to overcome by the patients. Consequently, administration of standard chemotherapeutics and platinum-based duplex regimens (e.g., cisplatin coupled with carboplatin, paclitaxel, or another cytotoxic drug) is also practiced for alleviating the lung tumor. An array of adverse side effects is also reported in lung cancer patients including ototoxicity, gastrointestinal toxicity, nephrotoxicity, hypersensitivity, neurotoxicity, myelosuppression, and increased resistance to chemotherapeutics^4–6^. Hence, there is an upsurging call for efficient, safe natural alternatives for mitigating lung cancer.

Over the past decade, plant-derived therapeutics became constructive agents for chemotherapy in contrast to the chemoresistance-inducing conventional standard drugs. Among them, phytopigments like carotenoids are vastly investigated for their abundant therapeutic potential including anti-cancer activity^7^. Interestingly, substantial epidemiological discoveries and the emerging concept of chemoprevention through diet paved the path to the exploration of apocarotenoids, the breakdown products of carotenoids. Carotenoids and apocarotenoids are well-known for their plethora of pharmaceutical applications including their potential to alleviate lung carcinomas^8^. Likewise, bixin is a prominent apocarotenoid extracted from the seed arils for *Bixa orellana* known as a food colorant and widely explored for its magnificent colouring utilities^9,10^. Despite these industrial applications, bixin is reported to have anti-aging, adipaging, nephroprotective, anti-tumor, and hypoglycemic properties^11–14^. Nonetheless, the anti-tumor potential of bixin is not explored in depth in the ailment of lung cancers.

In the current study, we reported the cytotoxic potential of bixin in A549, pulmonary alveolar carcinoma cell lines targeting BAX/BCL-2 and Cyclin D1 for the first time. Needless to mention, human lung carcinogenesis is an intricate mechanism at the molecular level, with a wide range of oncogenes. It is observed that the loss of apoptotic control permits the lung tumor cells to endure longer and provides additional time for the buildup of mutations which eventually augments the invasiveness for tumor progression, inducing angiogenesis, uncontrolled cell proliferation, and differentiation. The mitochondrion-centered intrinsic apoptotic pathway involving key molecular targets like BAX, BCL-2, and caspases is often targeted to activate apoptosis to suppress lung tumor growth^15^. Besides, numerous reports underlined the CCND1 A870G polymorphism and overexpression of cyclin D1 in NSCLC patients. Cyclin D1 overexpression is regarded as one of the factors in the malignant transformation in the lung and other tissues^16^. Hence, we performed a series of *in-vitro* assays in pulmonary adenocarcinoma cell lines (A549) followed by the *in-silico* analysis to delve into the molecular mechanism of bixin in alleviating lung cancer.

## Results and discussion

### Inhibitory effect of bixin on cell growth

**Fig. 1:**
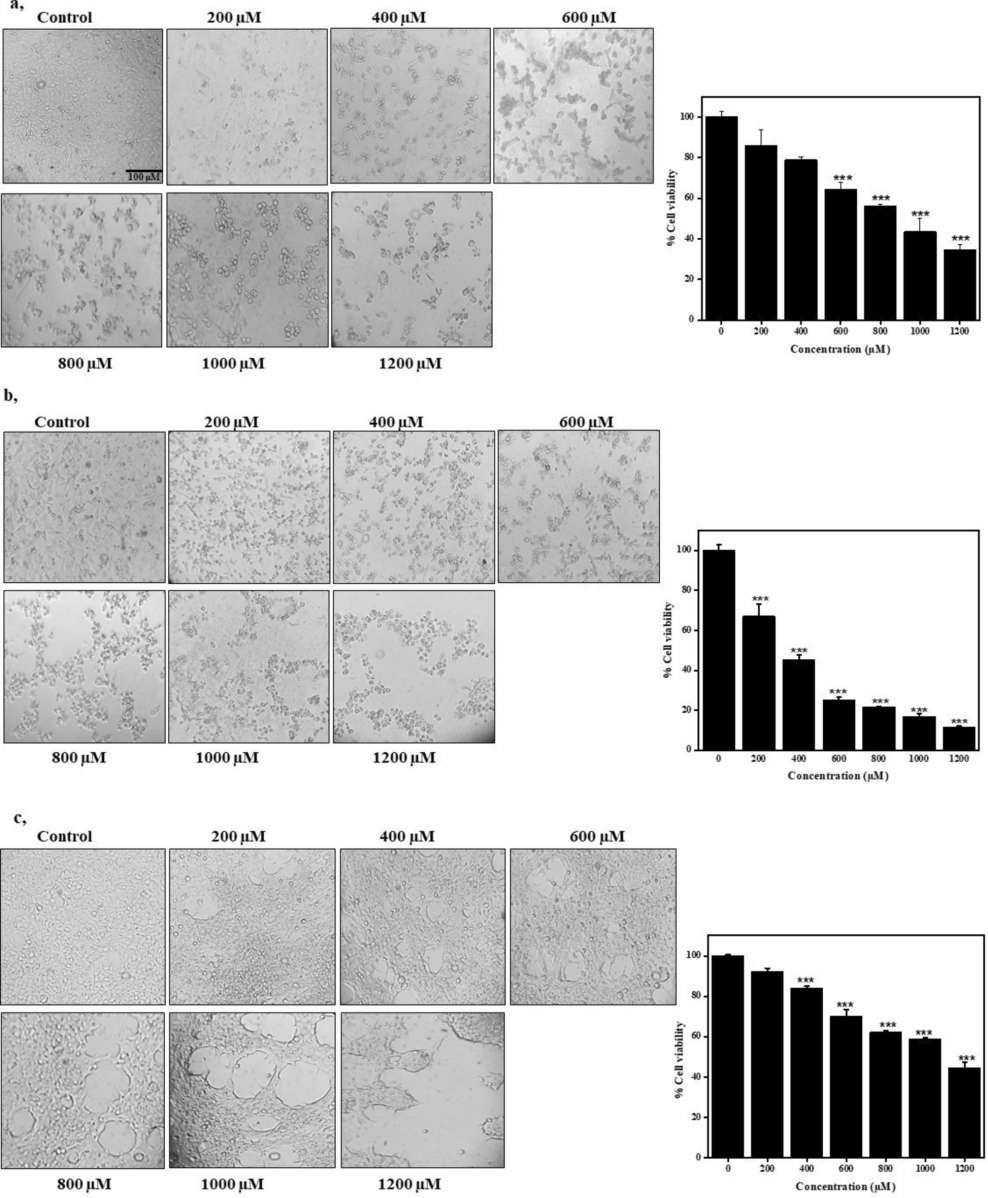
Bixin inhibited the proliferation of A549 lung cancer cell line and HEK-293 cell lines. (**A**) Microscopic images of A549 treated with gradient concentration of bixin (0–1200 µM) at 24 h. Graph showing the MTT results of A549 treated with gradient concentrations of bixin (0–1200 µM) at 24 h. (**B**) Microscopic images of A549 treated with gradient concentration of bixin (0–1200 µM) at 48 h. Graph showing the MTT results of A549 results treated with gradient concentration of bixin (0–1200 µM) at 48 h. (**C**) Microscopic images of HEK-293 treated with gradient concentration of bixin (0–1200 µM) at 24 h. Graph showing the MTT results of HEK-293 treated with gradient concentration of bixin (0–1200 µM) at 24 h. Scale for all the images is 100 µm. Data are presented as mean ± SD, n = 3, ***p < 0.001 compared to control.

The cytotoxicity imparted by bixin was evaluated on the A549 lung cancer cell line as well as HEK-293 normal cell lines in a dose-dependent manner. The cells were treated with a gradient concentration of bixin dissolved in DMSO ranging from 0 to 1200 µM. Considerable changes were observed in the cell morphology of A549 on the administration of a high concentration of bixin within 24 hours as shown in Fig. 1A. After 48 hours, adherent cells lost adherence to the substrate leading to the rounding of cells and lowered cell count even in the lowest concentrations as shown in Fig. 1B. The cytotoxicity was observed to be considerably lower in HEK 293 cell with an IC50 of 1109.89 µM (Fig. 1C). The IC50 of bixin in A549 cells was estimated to be 899.24 µM. The concentrations of bixin proposed for further investigation were 400 µM, 800 µM, and 1200 µM.

### Bixin curbing the migration of A549 cells

**Fig. 2:**
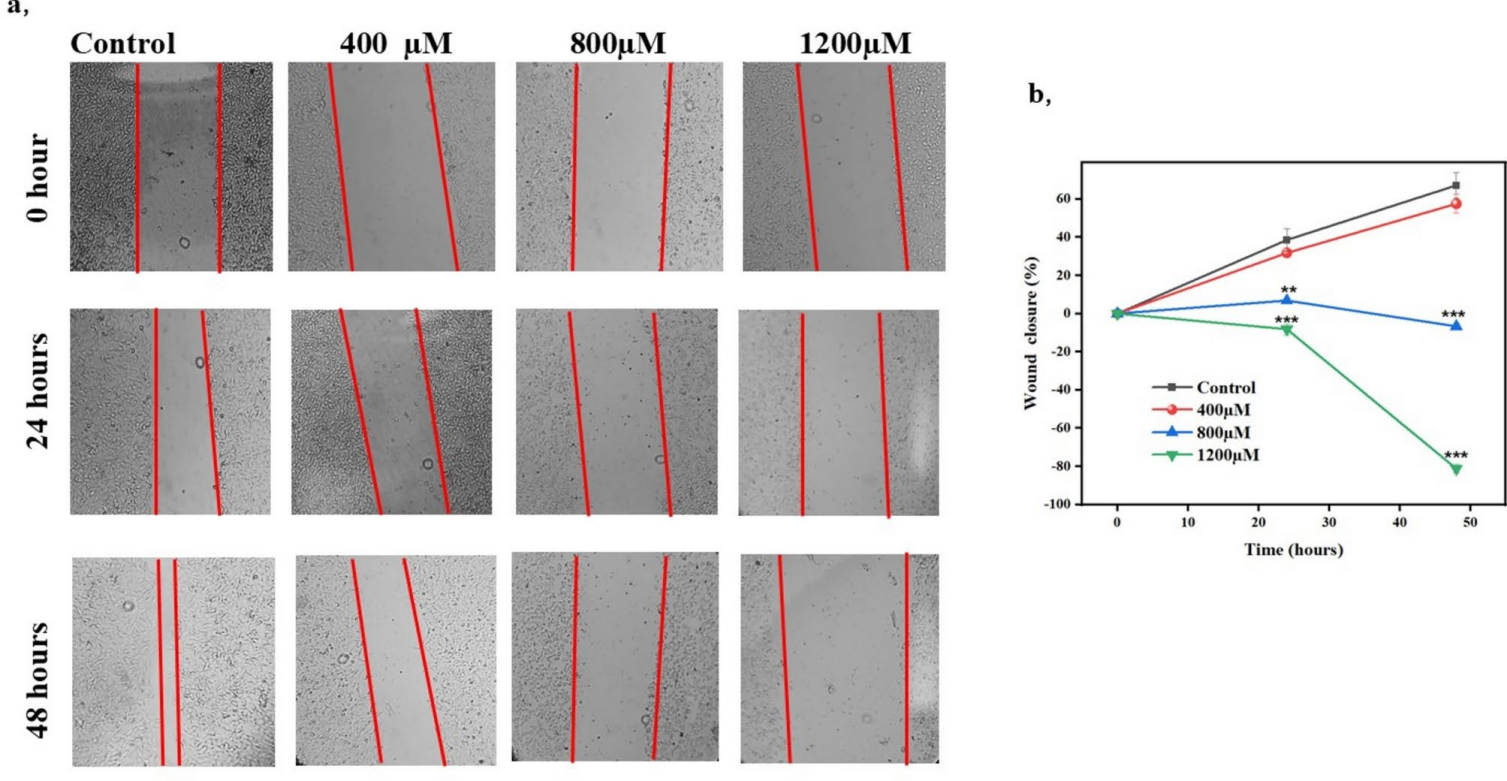
(**A**) Effect of bixin on cell migration of A549 at different concentrations of 400 μM, 800 μM and 1200 μM. Scale bar is 100 µm. (**B**) Wound closure percentage in the time period of 48 h. Data are presented as mean ± SD, n = 3, *p ≤ 0.05, **p < 0.01, ***p < 0.001 compared to control.

Tumor metastasis is one of the foremost concerns observed in lung sarcoma patients and wound healing assay was performed to assess the effect of bixin on migration of A549 lung cancer cell lines. Cell margins were photographed immediately after the scratch and at regular intervals (24 and 48 hours). The migration effect was pronounced as seen on Fig. 2. The movement of cells was observed individually in control and 400 µM bixin-treated cells in 24 hours. Expectedly, the cell migration rate was higher, and the wound was nearly closed in untreated cells. In contrast, the mobility of cells was largely affected in 800 µM and 1200 µM bixin-treated cells. The morphology of the cells was altered considerably, and the wound area increased in 1200 µM bixin-treated cells within 48 hours.

### Apoptotic morphological changes induced by bixin

**Fig. 3:**
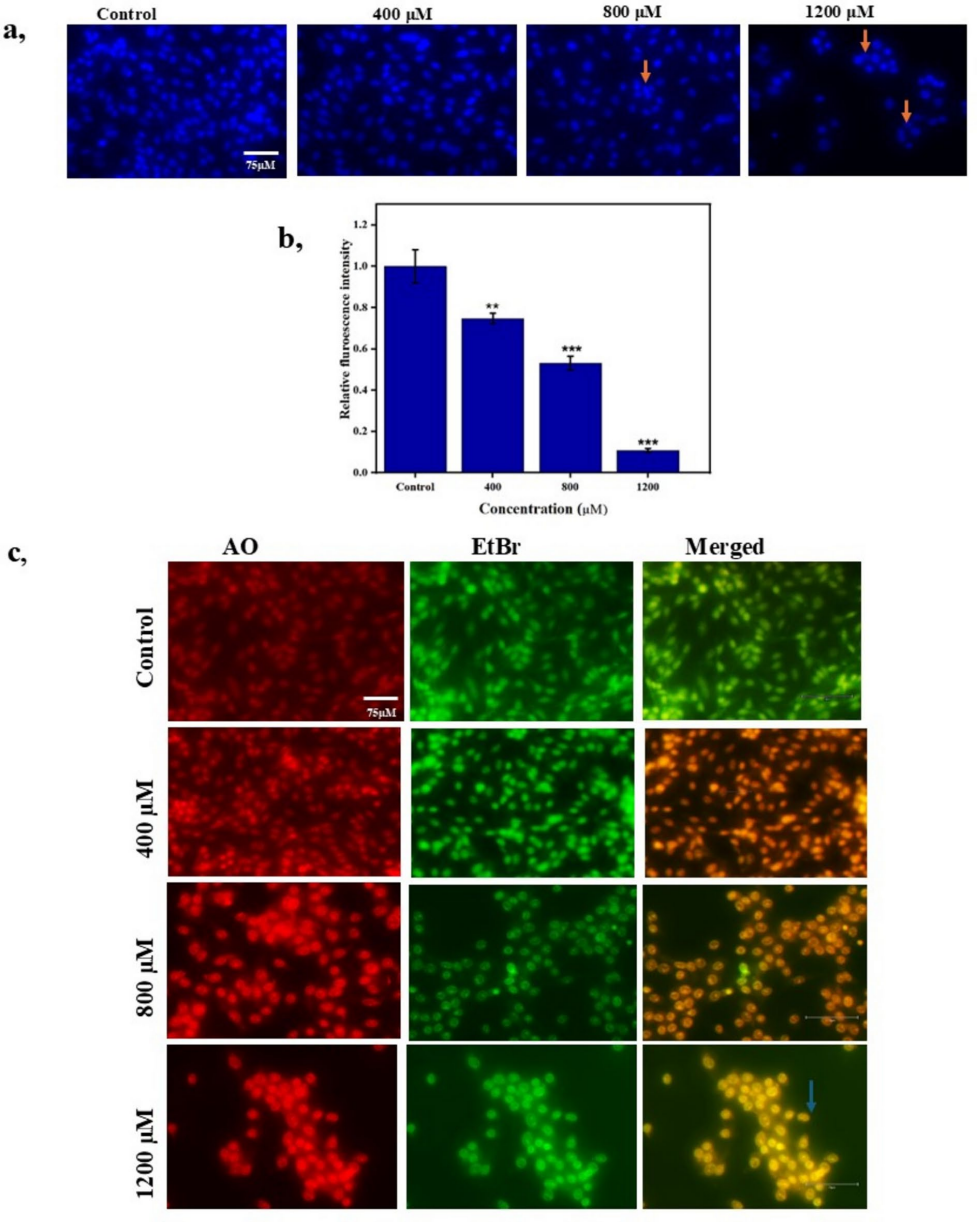
Effect of bixin on A549 at different concentrations of 400 μM, 800 μM and 1200 μM. (**A**) Fluorescent images representing apoptotic morphology evaluated by DAPI. Scale for images is 75 µm. (**B**) Relative fluorescence indicating the reduction in the number of cells at different concentration. Data are presented as mean ± SD, n = 3, **p ≤ 0.01, ***p < 0.001 compared to control. (**C**) Fluorescent images representing apoptotic morphology evaluated by Acridine Orange-Ethidium bromide staining. Scale for images is 75 µm.

Apoptosis is a highly regulated program involving several signaling cascades characterized by morphological and biochemical changes like nuclear and cytoplasmic condensation, fragmentation of DNA, changes in mitochondrial membrane potential, and generation of apoptotic bodies^17^. DAPI (4',6-diamidino-2-phenylindole), a fluorescent compound permeates the cell membranes and selectively binds to the minor groove of A/T-rich DNA regions of nuclei emitting a blue fluorescence. DAPI staining is widely utilized in cancer cells including A549 cells to examine the morphological changes in nuclei^18^. The nuclear morphology was observed to be intact in control and 400 µM bixin-treated cells. Interestingly, the condensed and fragmented nuclei were observed in 800 and 1200 µM bixin-treated cells. Besides, the nuclei with apoptotic morphology increased with increasing concentration of bixin as shown in Fig. 3a. The decrease in fluorescence intensity was owing to the decrease in the number of nuclei on bixin-exposed cells (Fig. 3b).

The dual staining approach utilizing Acridine Orange (AO)/Ethidium Bromide (EB) is widely employed to assess the apoptotic morphology induced by the anti-cancer agents^19^. Profound morphological changes including nuclear shrinkage, and chromatin condensation were observed in 800 and 1200 µM bixin treated A549 cells. Late apoptotic cells with red and orange nuclei were observed in the three administrated concentrations of bixin due to the uptake of EO through the damaged membrane. In contrast, early apoptotic cells were also observed with green nuclei in the 800 µM treated cells as seen in Fig. 3c. The number of stained cells was also decreased considerably owing to the apoptotic effect of bixin.

### Bixin lowered the mitochondrial membrane potential

**Fig. 4:**
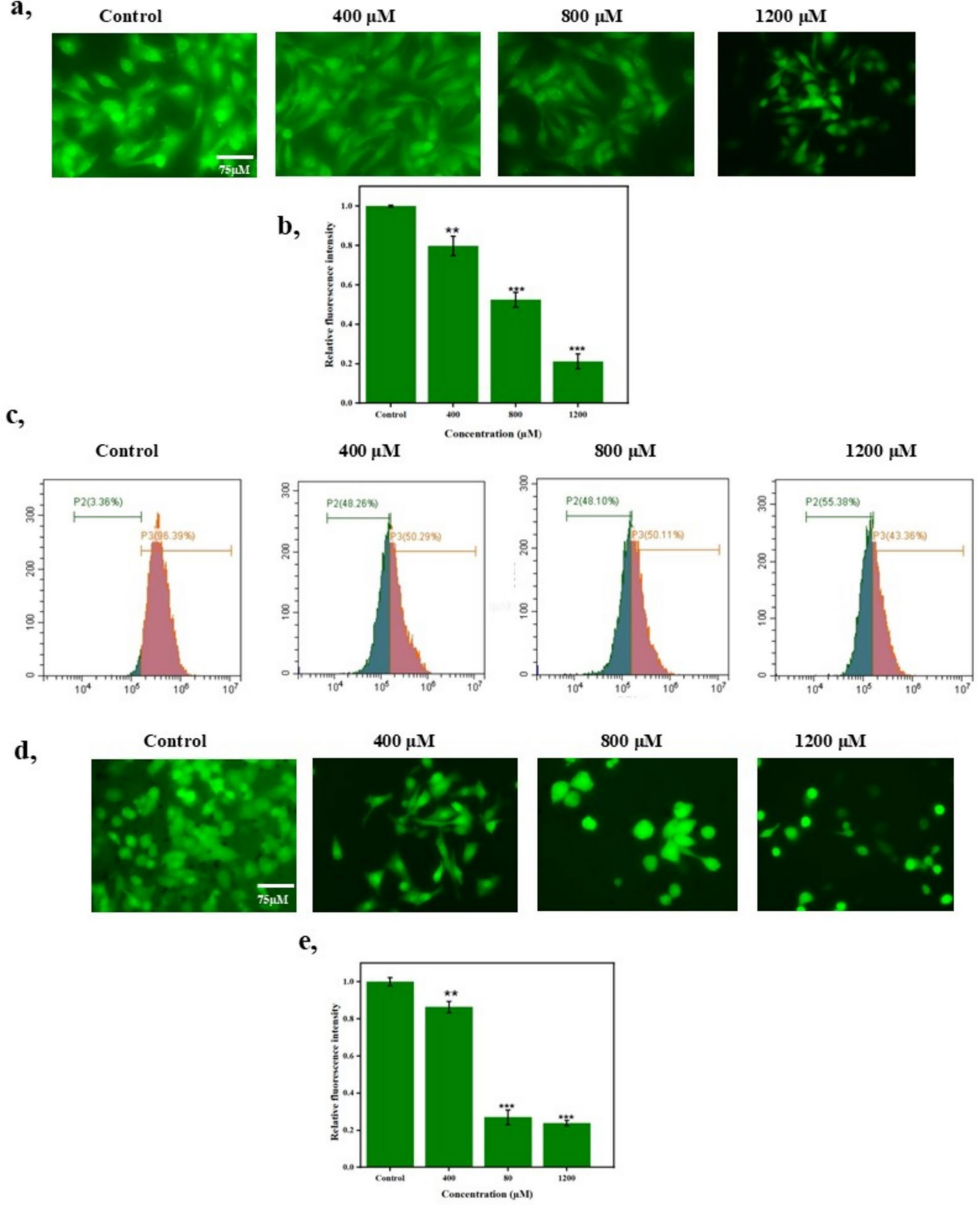
(**A**) Images representing decrease in fluorescence as indicative of reduction in MMP evaluated by Rhodamine 123 at 488/535 nm. Scale for images is 75 µm. (**B**) Relative fluorescence indicating the reduction of MMP. Data are presented as mean ± SD, n = 3, **p ≤ 0.01, ***p < 0.001 compared to control. (**C**) Effect of bixin on mitochondrial membrane potential of A549 at different concentrations of 400 μM, 800 μM and 1200 μM analyzed through flow cytometry using Rhodamine 123. The reduction in the percentage of fluorescence emitted by the cells is an indicative of decrease in mitochondrial membrane potential as seen in apoptotic conditions. (**D**) Images representing decrease in fluorescence as indicative of reduction in ROS evaluated by DCFH-DA. Scale for images is 75 µm. (**E**) Relative fluorescence indicating the reduction in the ROS. Data are presented as mean ± SD, n = 3, **p ≤ 0.01, ***p < 0.001 compared to control.

The proton pumps (Complexes I, III, and IV) generate the mitochondrial membrane potential (ΔΨ_m_) which is an integral component in the energy storage during the oxidative phosphorylation process in cells. The ΔΨ_m_ forms the transmembrane potential of hydrogen ions along with the proton gradient to produce ATPs essential for physiological and biochemical activities. However, cancer cells exhibit an abnormally high ΔΨ_m_ owing to their aggravated invasive and metastasis nature^20^. Furthermore, the loss of mitochondrial membrane integrity will reduce the ΔΨ_m_, an early event in apoptosis. We assessed the potential of bixin in curtailing ΔΨ_m_ in A549 cells through Rhodamine-123 labeling with the aid of a fluorescent microscope and flow cytometry^21^. This cationic fluorescent dye distributes on par with the negative ΔΨ_m_. As shown in Fig. 4a, b, and c, the fluorescence intensity decreases in treated cells due to the loss of ΔΨ_m_. Figure 4c shows the gradual reduction in fluorescent cells count as an index of loss of ΔΨ_m_ within 4 hours of treatment. The proportion of fluorescent cells reduced from 96.39 to 43.36% as the concentration increased, showing damage in the mitochondrial membrane.

### Bixin curbed the ROS production in A549

ROS, defined as the molecules involved in the transfer of electrons from reactive oxygen, plays a crucial role in tissue homeostasis, cellular signaling, differentiation, and survival. Augmented ROS accumulation, as a consequence of metabolic disturbances and signaling aberrations, leads to carcinogenesis and subsequent malignant progression via stimulating gene mutations and oncogenic signaling^22^. Interestingly, bixin is known for its impeccable antioxidant nature with the highest potential of 0.94 V among the carotenoids^23^. We explore the ROS-curbing property of bixin in A59 cells through DCFH-DA labelling aided by fluorescent microscopy^24^. As shown in Fig. 4d and e there was a considerable reduction in fluorescence intensity and thus the ROS production as the concentration of bixin increased.

### Quantification of bixin induced apoptosis through Annexin V-FITC/ PI

The apoptotic inducing potential of bixin was further substantiated by employing Annexin V-FITC/ Propidium iodide (PI) staining and the subsequent measurement of the count of cells in various apoptotic stages. PI is largely applied in conjunction with Annexin V to determine whether the cells are viable, apoptotic, or necrotic by means of disparities in plasma membrane integrity and permeability, which occurs in late apoptosis and necrosis. Whilst Annexin V exhibits a strong, Ca2^+^-dependent affinity for phosphatidylserine (PS) residues which are only exposed in apoptotic cells and hence known as a probe for detecting apoptosis^25^.

**Fig. 5:**
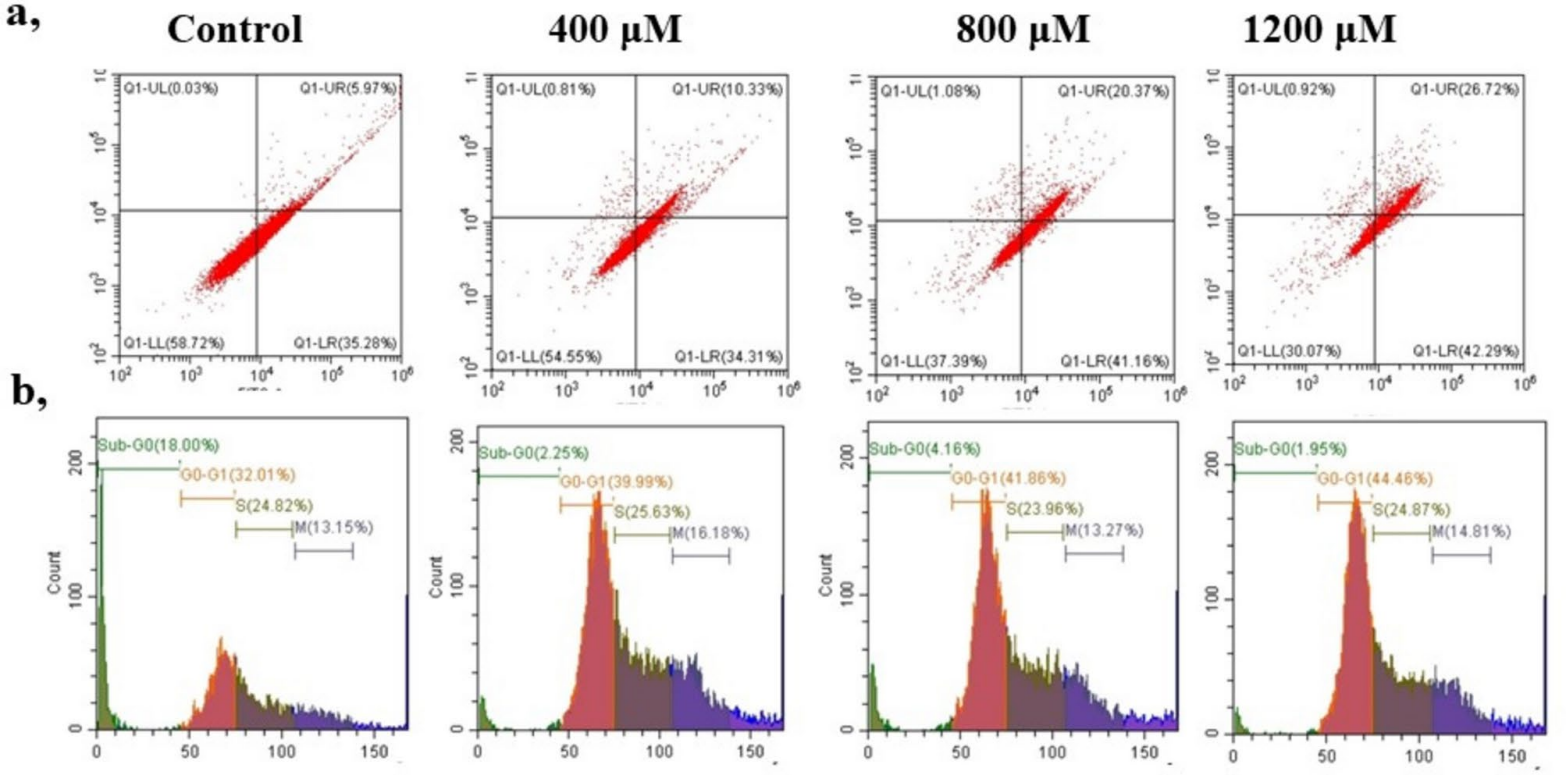
(**A**) The Effect of bixin on apoptosis of A549 lung cancer cell line at different concentrations of 400 μM, 800 μM and 1200 μM analyzed through flow cytometry using annexin V-FITC/ propidium iodide. (**B**) Impact of bixin on cell cycle progression of A549 lung cancer cell line at different concentrations of 400 μM, 800 μM and 1200 μM analyzed through flow cytometry.

As shown in Fig. 5a the quadrants denote living cells (Annexin V^−^PI^−^), early apoptotic (Annexin V^+^PI^−^), late apoptosis (Annexin^+^PI^+^), and the necrotic (Annexin V^-^PI^+^) stages. The late apoptotic cells increased to 42.29 % in 1200 µM as compared to the control. Moreover, the late apoptotic cells were 26.72 % and live cells were only 30.07 % in the highest dose of bixin treated cells underlining its apoptosis-inducing nature.

### Effect of bixin on cell cycle progression

We further examine the stage of cell cycle progression where bixin is imparting its apoptotic potential through flow cytometric analysis as reported earlier^26,27^. The cell cycle is the conserved mechanism by which eukaryotic cells replicate themselves. Transmission of genetic material from one cell to the next entails genome replication happening in the S-phase and its segregation to the two new daughter cells during mitosis or M-phase. In a normal cell cycle, the S-phase is generally preceded by the M-phase. There are two preparatory gaps in between; G1 and G2. G1 separates M from S, and G2 is between S and M. Interestingly, the manipulation of the cell cycle may suppress or stimulate an apoptotic response depending upon the cellular context^28^.

As shown in Fig. 5b, the proportion of cells in the G0/G1 phase increased gradually with an increase in the concentration of bixin. Besides, there was a reduction in the proportion of Sub G0 cells with an increase in treatment dosage. The percentage of cells in the M phase and Sub-Go didn’t show any significant variations as compared to the other cell cycle stages. The accumulation of cells in Go/G1 underlines that the bixin arrests cell cycle progression at the G0/G1 phase and curtails the transition to the S phase.

### Bixin promoted the activity of Caspase 3/7 and Caspase 9

**Fig. 6:**
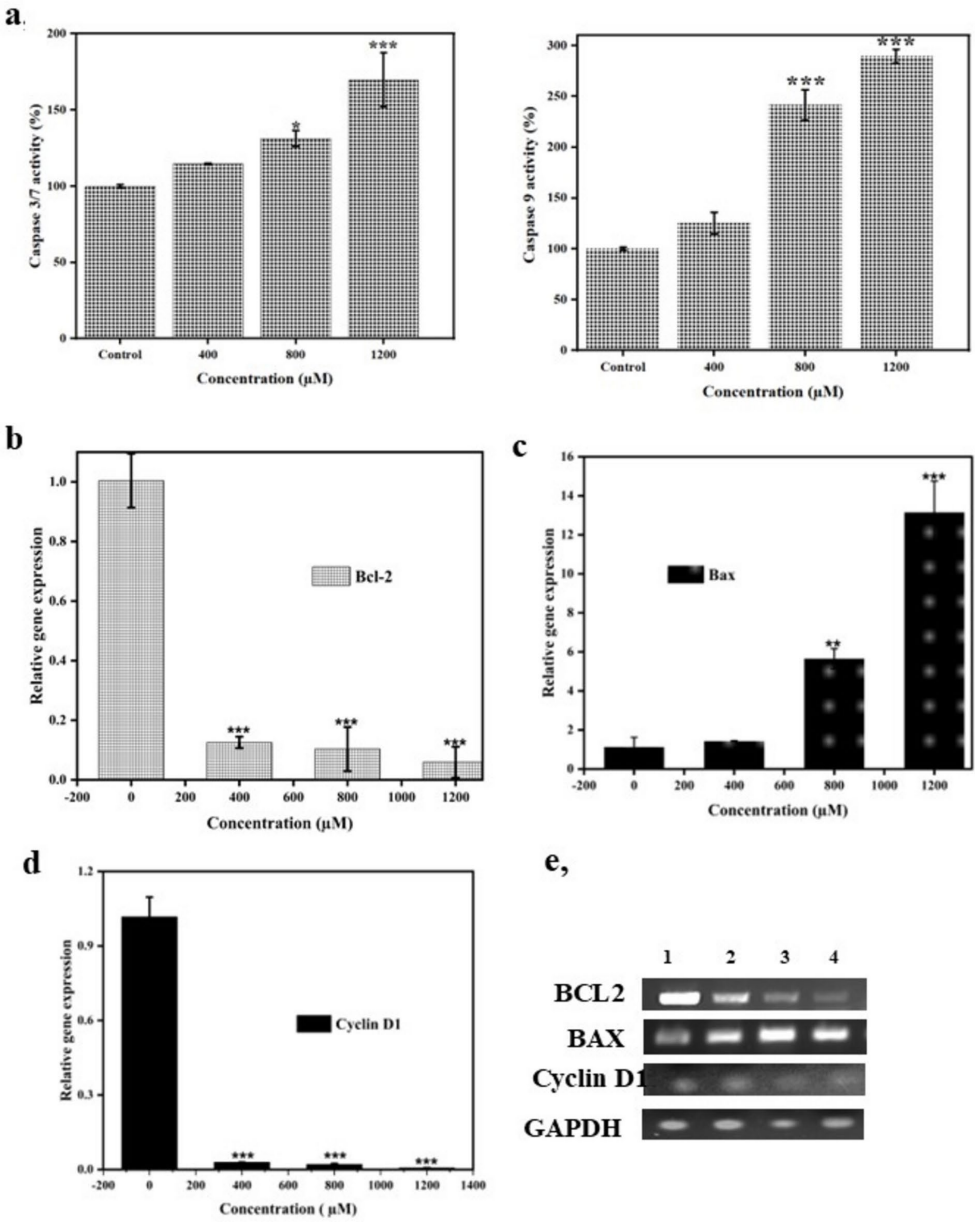
(**A**) Bixin enhanced the activity of caspase 3/7 and Caspase 9 of A549 lung cancer cell line at different concentrations of 400 μM, 800 μM and 1200 μM. (**B**) Effect of bixin on expression of B. BCL-2 (**C**) BAX and (**D**) Cyclin D1/CCND1 of A549 lung cancer cell line at different concentrations of 400 μM, 800 μM and 1200 μM analyzed through RT-PCR. *p ≤ 0.05, **p ≤ 0.01 and ***p ≤ 0.001 compared to control. (**E**) Gel images showing the expression of genes in control and varying concentrations of bixin. Lane 1, lane 2, lane 3 and lane 4 are control, 400 μM, 800 μM and 1200 μM respectively.

Intrinsic apoptosis is defined as a mitochondrion associated cell death affecting mitochondrial outer membrane permeabilization (MOMP) and developing apoptosomes leading to the activation of caspase-9 and succeeding activation of other effector caspases^29^. Intrinsic apoptosis is highly regulated by BCL-2 and caspases, where caspases are a family of endoproteases synthesized as zymogens. The intrinsic pathway commences by the release of cytochrome *c* to the cytoplasm from the mitochondrial intermembrane spaces. Further, cytochrome *c* interacts with Apaf-1 (apoptotic protease activating factor-1), an adaptor protein forming the heptameric structure of the apoptosome complex, which further activates caspase-9. Activated caspase-9 then directly cleaves and activates effector caspases. Caspase 3, a well-known effector caspase, is later cleaved by activated caspase 9, directing the apoptotic events^30^. Caspase 7 has been proven to play a significant role in the execution phase of apoptosis aiding the detachment of cells from extracellular matrix^29^. We performed caspase assays to evaluate the involvement of caspases in bixin induced apoptosis. As shown in Fig. 6a. bixin enhanced the expression of caspase 3/7 and caspase 9 in a dose-dependent manner. The caspase 3/7 activity was increased to more than 150% as compared to the control. While the caspase 9 activity was also high in 1200 µM treated cells. Hence, the results suggest that bixin imparts cytotoxicity in A549 lung cancer cells through mitochondrion-mediated intrinsic apoptotic pathways via targeting effector caspases.

### Impact of bixin on genes associated with apoptosis and cell cycle progression

Effectual suppression of cancer cells by programmed cell death or apoptosis has been a stronghold and target of clinical cancer therapy for many decades. The BCL-2 (B-cell lymphoma 2) protein family predominantly influences whether a cell moves to apoptosis and therefore plays critical roles in development, tissue homeostasis, and immunity. Abnormalities in BCL-2 family proteins are observed in NSCLC widely^31^. The BCL-2 family consists of a highly conserved BH domain forming its basis of function. The BCL-2 family of proteins is majorly classified into three subfamilies: BCL-2 and BCL-XL (anti-apoptotic proteins), BAX and BAK (pro-apoptotic proteins), and BAD and BID, the BH3 only proteins. In the current study, we explore the effects of bixin on the expression of BCL-2 and BAX. BCL-2 can impede apoptosis by suppressing the activity of caspase- 3,6,7, 9 and by regulating the Ca^2+^ for prolonged cell survival. On the other hand, activated BAX binds to the mitochondrial membrane generating MOMP and subsequent apoptotic events^32^. Interestingly, the bixin upregulated the expression of pro-apoptotic BAX and downregulated the expression of antiapoptotic protein BCL-2 as shown in Fig. 6b and c in a dose-dependent manner. BCL-2 expression was reduced to a negligible value and Bax expression was upregulated highly in 800 µM and 1200 µM treated cells as shown in Fig. 6b and c.

Moreover, we also explore the effect of bixin on cyclin D1 since bixin arrests the cell cycle at the G0/G1 phase. Cyclin D1 is over-expressed in solid tumors particularly non-small lung carcinoma^33^. Cyclin D1 overexpression is regarded as a central factor in the malignant transformation in the lung and other tissues. It has been identified that smoking triggers nuclear accumulation of cyclin D1 in the human bronchial epithelium which stimulates unrestrained proliferation in normal human cells, facilitating the advancement of invasive lung cancer^16^. Cyclin D1 overexpression may occur due to CCND1 amplification or chromosomal rearrangements, and impaired degradation of the protein in tumor cells. Besides, Cyclin D1 regulates the transition from G1 to S phase by being an allosteric regulator of CDK 4 and 6^34^. As shown in Fig. 6d, RT-PCR results evidenced that bixin has downregulated the expression of CCND1 gene encoding cyclin D1 to a negligible value. Hence, bixin arrests the cell cycle at GO/G1 by suppressing the expression of cyclin D1. Amidst the pursuit of synthetic cyclin D1 inhibitors, bixin is proposed for the first time as a natural alternative.

### Interaction of bixin with molecular target proteins

**Fig. 7:**
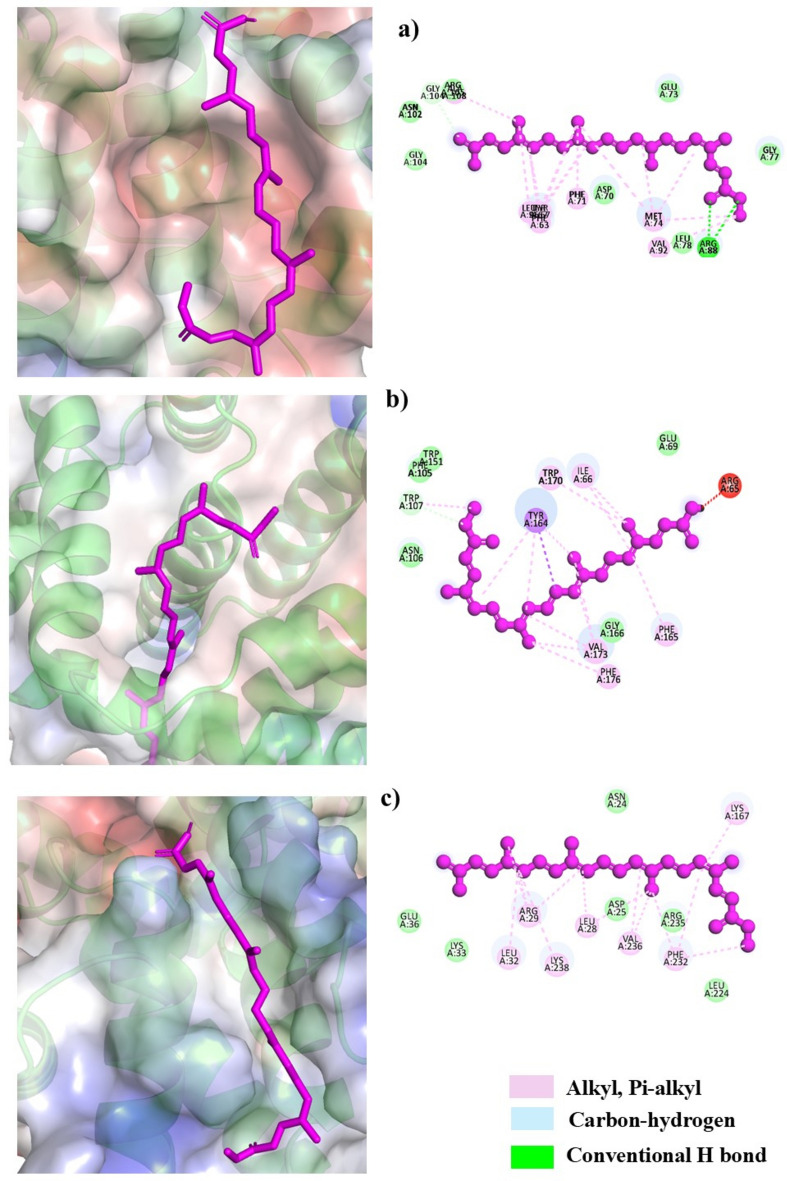
The docking poses and the 2D images of the interaction of bixin with (**A**) BCL-2, (**B**) BAX and (**C**) Cyclin D1.

The molecular docking results showed that bixin interacts with BCL-2, BAX, and Cyclin D1 by affinities of − 6.7 kcal/mol, − 7.0 kcal/mol, and − 7.1 kcal/mol respectively. Bixin binds to the electronegative sites of BCL-2 and interactions through van der Waals, alkyl, and Pi-alkyl interactions. A strong hydrogen is also formed with ARG88 (Fig. 7a). The van der Waals, carbon-hydrogen bond interactions, alkyl, pi-alkyl, and pi-sigma bond at TRP170 were observed in BAX-bixin as shown in Fig. 7b. In the context of cyclin D1, alkyl and pi-alkyl bonds were observed predominantly (Fig. 7c).

**Fig. 8:**
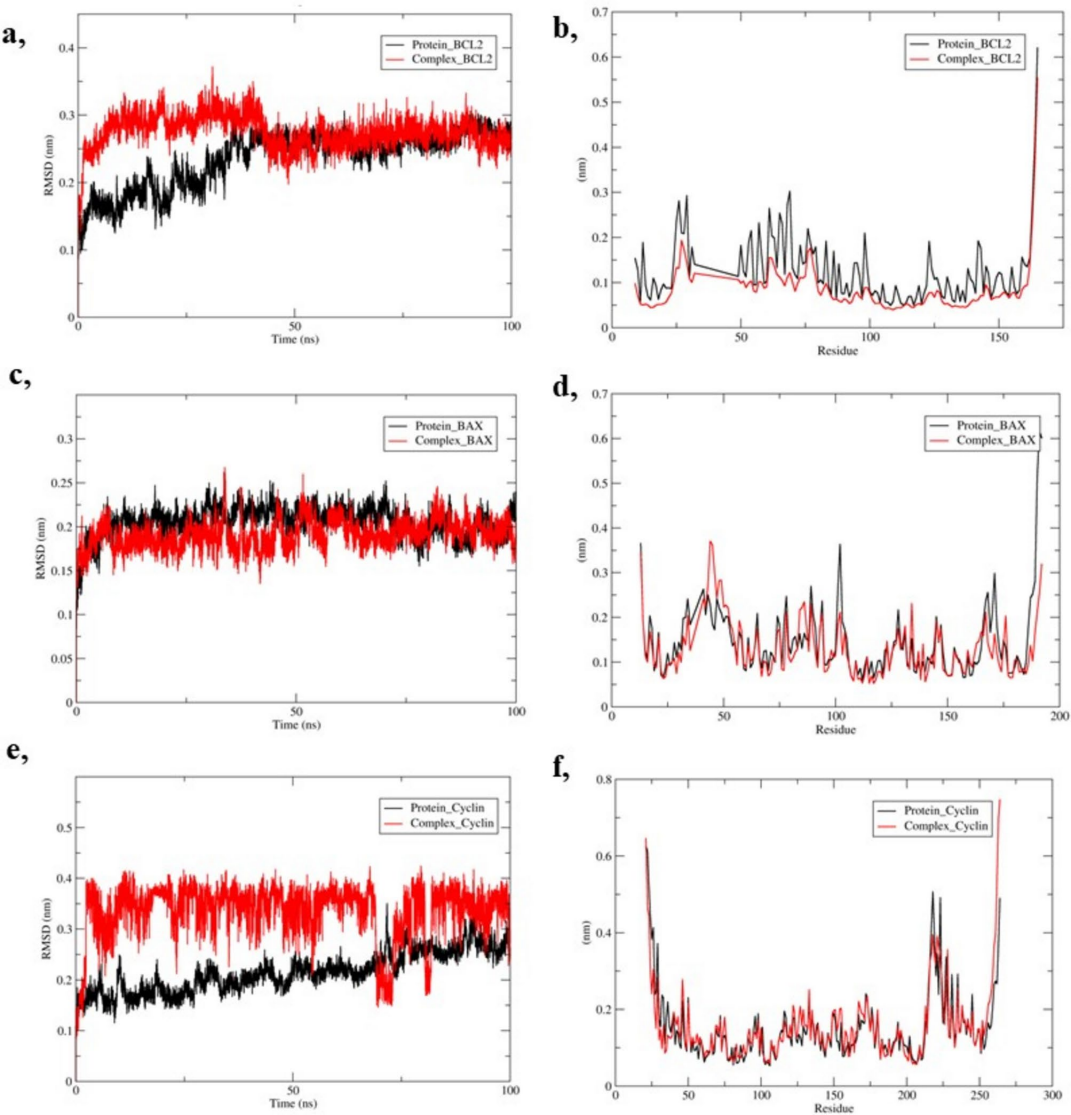
The RMSD profiling of the interaction of bixin with (**A**) BCL-2, (**C**) BAX and (**E**) Cyclin D1. The RMSF analysis of interaction of bixin with (**B**) BCL-2, (**D**) BAX and (**F**) Cyclin D1.

The RMSD profiling provide insights into the stability and structural convergence of proteins in interaction with the ligand. The trajectory for BCL-2 and the complex was observed to fluctuate to 45ns and was found equilibrated throughout the simulation at 0.25 nm. The complex showed fluctuation up to 0.35 nm and it was about 0.25 nm for the protein, as shown in Fig. 8a. In the case of BAX and complex, significant fluctuations were not observed and showed a very similar trajectory from 75 ns. Both the complex and BAX merged at the initial ns of the simulation and demonstrated minor fluctuations (Fig. 8c). The RMSD analysis of cyclin D1 complexed with bixin showed that the complex and apoprotein stabilized around 75 ns and the complex showed fluctuations of 0.1 nm to 0.2 nm throughout the simulation as compared to apo-protein (Fig. 8e).

The positional fluctuations in each amino acid residue were observed in the RMSF analysis. Interestingly, fluctuations up to 0.3 nm were observed from 50 to 150 amino acid residues in BCL-2. The interaction with the bixin has limited these fluctuations throughout the simulation, underlining the effects of bixin on BCL-2 (Fig. 8b). In bixin-BAX complex, the ligand flexibilized at the residues before 50 up to 0.4nm. The fluctuations were observed to be less for the complex throughout the simulation (Fig. 8d). Positional fluctuations were observed more from 200 to 225 amino acid residues in cyclin D1. However, the interaction with bixin lessened the fluctuation indicating the modifications induced by bixin (Fig. 8f). The rg, SASA and hydrogen bond analysis of the bixin-protein complex is provided in Supplementary Fig. S1.

## Discussion

The global occurrence and mortality rate of lung cancer is tremendously rising every year irrespective of age, race and socioeconomic status. Over the year, a multitude of therapeutics have been developed to curb the growing menace. Later, natural products were established as successful chemotherapeutics owing to their increased effectiveness in clinical and sub-clinical investigations^35^. Interestingly, phytopigments including carotenoids are proven to have effects on lung carcinomas in *in vivo* and *in vitro* research^8^. However, bixin, a highly valuable apocarotenoid was not well explored in lung cancer research. Here, we identified the potential of bixin in ameliorating the NSLC through an integrated *in vitro* and *in silico* analysis.

In our study, bixin imparted toxicity at concentrations above 600 µM inducing considerable apoptotic morphological changes. Besides, invasiveness of lung cancer is a factor hindering effective therapy. Hence curtailing the migratory effects of tumor cells plays an undeniable role in alleviating the detrimental effects of metastasis as explored in previous studies^6,36^. We observed the anti-migratory effect of bixin in A549 even at the lowest concentration of 400 µM. The extensive fluorescent labelling assays using DAPI, AO-EtBr further underlined the potential to induce apoptosis in A549 lung cancer cell line. Similar apoptotic morphological characteristics of nuclear fragmentation and chromatin condensation have been reported with the assistance of a fluorescence microscope in A549 lung cancer cell lines^21,37^. Furthermore, bixin lowered the mitochondrial membrane potential inducing apoptosis in lung cancer cell lines. Other natural products like gracillin from *Reineckia carnea* have shown similar effects by reducing MMP in A549 cells and stimulated mitochondrial apoptosis^38^.

The antioxidant nature of bixin was also significantly observed in the study in contrast to the pro-oxidant effects of natural molecules in cancer cell lines as previously reported. We also further corroborated and quantified the apoptosis induced by bixin through the Annexin V/PI flow cytometry analysis. Similar to bixin, there are also Annexin V/PI reports of plant extracts and their secondary metabolites inducing apoptosis in lung cancer cell lines. For instance, *Swertia chirayita* curbs the growth of A549 by inducing apoptosis and suppressing JAK1/STAT3 expression^39^.

Furthermore, we noticed bixin arrested the cell cycle progression of A549 at G0/G1 phase. As per previous studies, bixin also arrested the Go/G1 phase in B16 melanoma cell lines^40^ and leukemia K562 cells^41^ substantiating the potential of bixin to halt the cell cycle progression. Obstruction of the cell cycle stage at the G1 phase is also perceived as the reason for the apoptosis induction in tumor cell lines by the bixin. Furthermore, p53-initiated cell cycle arrest is an important event in the regulation of G1/S checkpoint. p53 activates the p21 protein, a vital cell cycle D inhibitor that can bind to the Cyclin-CDK complexes and thus inhibit kinases activity. In G1 phase, CDK4/6-Cyclin D complexes, which causes cell cycle arrest at the G1 phase. Besides, it also induces mitochondrial intrinsic pathway eventually causing apoptosis.

Taking into account of the apoptosis instigated by bixin, we further validated the molecular targets of bixin through gene expression studies and caspase assays. We have observed that bixin instigated a mitochondrial mediated intrinsic apoptosis to limit lung cancer proliferation. As already stated, intrinsic apoptotic cell death involves mitochondrial outer membrane permeabilization followed by the release of cytochrome c, formation of the apoptosome with APAF1, and initiator caspase 9 leading to the activation of executioner caspase 3 and apoptosis. The intrinsic apoptotic pathway is triggered by numerous external and internal factors including oxidative stresses, irradiation, and treatment with cytotoxic drugs^42^. Proteins that are generally involved in intrinsic pathways other than caspases include Bcl-2 (B-cell lymphoma protein 2), Bcl-w (Bcl-2-like protein), Aven (Cell death regulator Aven), SMAC/DIABLO (Second mitochondrial activator of caspases/direct IAP binding protein with low PI), Nox (Phorbol-12-myristate-13-acetate-induced protein 1) and Myc (Oncogene Myc)^43^. Most importantly, BCL-2 is a target of several anticancer agents to curb tumor growth through intrinsic apoptosis^44^. Interestingly, bixin lowered the expression of BCL-2 and enhanced the expression of proapoptotic BAX. Additionally, bixin upregulated the activity of caspase 3/7 and caspase 9, key effector caspases in the intrinsic apoptosis. Similar mitochondrial mediated apoptosis has been reported by natural molecules like Plumbagin, a napthoquinone from *Plumbago zeylanica*^21^, Narigenin, a flavonoid from citrus fruits^45^, sesamin^46^ in the A549 lung cancer cell lines.

We also look into the effects of bixin on the expression of Cyclin D1/CCND1 since bixin curbed the cell cycle at G0/G1 phase. Interestingly, bixin lowered the expression of CCND1  cyclin D1. Unrestrained cell proliferation is a hallmark of cancer where cell cycle progression is not regulated. Cyclins (A, B, D, and E) and their associated cyclin-dependent kinases (CDKs 1, 2, 4, and 6) are the major cell cycle regulatory machines via phosphorylating cell cycle-related proteins to endorse the progression of cell growth^47^. Cyclin D1 along with cyclin-dependent kinase 4 (CDK) and CDK6, acts as a mitogenic sensor and integrates extracellular mitogenic signals and cell cycle progression. There are reports underlining the role of cyclin D1 in the proliferation and invasiveness of lung cancer. However, the reports of natural products targeting cyclin D1 in lung cancer are limited. Synthetic molecules like dihydroartemisinin have targeted AKT/GSK3β/cyclinD1 to induce apoptosis in lung cancer cell lines^48^ and cediranib arrested cell cycle and induced apoptosis via CDK4/cyclin D1 and CDK2/cyclin E^49^. We also assessed the interaction of bixin with the key molecular targets BCL2, BAX and Cyclin D1 through molecular docking and MDS. A stable interaction was observed in the analysis In a nutshell, we unravel the potential of bixin on pulmonary alveolar adenocarcinoma migration and proliferation via targeting BAX/BCL-2 and Cyclin D1. Ample in-vivo and clinical research can further fill the lacunae in promoting the anti-lung cancer potential of bixin. Comprehensively, bixin can be developed as a nutraceutical supplement considering its therapeutic role. Unfortunately, there was a fall in carotenoid research owing to the negative impact generated by beta-carotene in previous clinical trials of lung cancer. Principally, the current results will aid in exploring more carotenoids for the ailment of tumors, particularly lung cancer.

## Methods

### Reagents

Bixin [≥90.0% (HPLC)] was purchased from Sigma-Aldrich. All the cell culture reagents were procured from HiMedia, Mumbai, Maharashtra, India. DAPI, Rhodamine 123, DCFH-DA, Propidium Iodide (PI), Ribonuclease, TriZol reagent were purchased from Sigma-Aldrich, Bangalore, Karnataka, India. Cell culture tested Acridine-orange, Ethidium Bromide, Trition X-100 from HiMedia, Mumbai, Maharashtra, India. Annexin V-FITC apoptosis kit was procured from Invitrogen, India, Caspase 3/7, caspase 9 kits from Elabscience Biotechnology Inc. Texas, USA, and PrimeScript RT reagent Kit from Takara, Otsu, Shiga, Japan.

### Cell culture

A549 and HEK-293 cells were received from the National Center for Cell Science, Pune, Maharashtra, India. Cells were maintained in Dulbecco’s modified Eagle’s medium supplemented with 10% fetal bovine serum (FBS), penicillin (100 IU/ mL), and streptomycin (100 µg/mL) in a 5% CO_2_ incubator at 37 °C. Cells were passaged in subsequent intervals depending on their confluency.

### Anti-proliferative analysis

A549 cells and HEK-293 cells were seeded at a density of 1 × 10^5^ cells/well in 96 well plates. Bixin dissolved in 10% DMSO was administered at varying concentrations of 200 µM, 400 µM, 600 µM, 800 µM, 1000 µM, 1200 µM. MTT assay was performed after 24 hours to assess the cytotoxicity imparted by bixin in the two cell lines as per Anantharaman et al.^40^. The cytotoxic effects of bixin were evaluated for another 24 hours in A549 cells. The absorbance of formazan product formed in MTT assay was measured at 570 nm in a microplate spectrophotometer (BioTek Epoch, Agilent). The IC50 values of bixin in A549 and HEK-293 cells were later calculated. The concentration of bixin for further analysis was determined based on the IC50 value in A549 cell lines.

### Anti-migratory assay

A549 lung cancer cells (1 × 10^4^ cells/well) were seeded in 6 well plates and maintained at 37 °C in 5% CO_2_ incubator. Once the cells reached near confluency, the monolayer was scratched using a 200 µl pipette tip generating a gap in the center of the well. Wells were then washed twice with PBS to remove the floating cells. Cells were then exposed to different concentrations of bixin (400 µM, 800 µM, 1200 µM). The photomicrographs (10× objective) were taken at different time points (0, 24, 48 hours) from an inverted microscope (Olympus LS). The wound closure rate was analyzed using Image J 1.54d software by measuring the wounded area without cells in the fixed time intervals. The decrease in wound closure rate indicates an increase in anti-migratory effect.

### Determination of apoptotic changes in morphology

The morphological changes induced in A549 cells on exposure to bixin were analyzed using various staining techniques^50^. DAPI, a nuclear stain, was used to stain the cells as per manufacturer’s instructions. The A549 cells (1 × 10^4^ cells/well) treated with different concentrations of bixin (400 µM, 800 µM, 1200 µM) for 24 hours were fixed in wells using 70% ethanol. After PBS wash, DAPI solution (300nM) was added into cells and incubated for 5-10 minutes in the dark and visualized under a Fluorescence microscope (EVOS M5000, Invitrogen). The fluorescence intensity was quantified from the obtained images using Image J 1.54d software.

Additionally, the detection of apoptotic cell formation was examined using the combination of AO and EB^51^. The A549 cells were treated with different concentrations of bixin (400 µM, 800 µM, 1200 µM) for 24 hours and were washed with PBS. Cells were stained with AO-EtBr (100 µg/ml) for 10min in the dark. The stained cells were observed under a Fluorescence microscope (EVOS M5000, Invitrogen). The fluorescence intensity was quantified using Image J 1.54d software.

### Detection of mitochondrial damage

The changes in mitochondrial membrane potential were determined microscopically and by flow cytometry using Rhodamine-123 (Rh-123) which binds specifically to metabolically active mitochondria^50^. A549 cells treated with bixin (400 µM, 800 µM, 1200 µM) at different concentrations were labeled using 1 µg/ml of Rh-123 and incubated in the dark for 30 minutes. The stained cells were washed properly with PBS and observed under a Fluorescence microscope (EVOS M5000, Invitrogen). The fluorescence intensity was quantified in FP-8300 Spectrofluorometer (Jasco, UK).

For flow cytometry analysis, the treated cells were trypsinized and centrifuged to obtain cell pellets. The cells were further suspended in PBS and labeled with Rh-123 in the dark for 30 minutes. The treated samples were examined in CytoFlex S and results were analyzed in CytExpert (Beckman Coulter).

### Detection of ROS generation

Generation of ROS was measured with 2ʹ,7′-DCFH-DA (Dichloro-dihydro-fluorescein diacetate), an oxidation-sensitive fluorescent probe. The deacetylated form, DCFH-DA was oxidized in the presence of ROS to highly fluorescent DCF. The A549 cells treated with bixin at different concentrations (400 µM, 800 µM, 1200 µM) were rinsed with PBS and incubated with DCFH-DA for 30 min in the dark. The cells were washed with PBS and visualized in a Fluorescence microscope (EVOS M5000, Invitrogen). The fluorescence intensity was quantified in FP-8300 Spectrofluorometer (Jasco, UK)

### Cell apoptosis assay

Apoptotic cells are examined by flow cytometry using eBioscience Annexin V-FITC apoptosis kit (Invitrogen, India). The experiment was performed as per the manufacturer’s instructions. Concisely, A549 lung cancer cells were seeded at a density of 2 × 10^5^ cells in 6-well plates. Cells were administered with different concentrations of bixin (400 µM, 800 µM, 1200 µM) for 4-5 hours and washed gently in PBS. Cells were resuspended in 1X binding buffer followed by the addition of Annexin V-FITC. These cells were incubated for 10 minutes at room temperature and later washed and resuspended in 1X binding buffer. PI (20 µg/ml) was added to the treated samples and examined in CytoFlex S and results were analyzed in CytExpert (Beckman Coulter).

### Cell cycle assay

Cell cycle analysis by the quantification of DNA content was performed using Propidium Iodide (PI), a DNA-binding dye^26^. Briefly, A549 cells were seeded at a density of 1 × 10^4^ cells in 6-well plates. Cells were administered with different concentrations of bixin (400 µM, 800 µM, 1200 µM) for 8 hours and later the trypsinized cells were fixed in 70% ethanol overnight. Further, cells were treated with 100 µg/ml of Ribonuclease. Cells were stained using a mixture of Triton X-100 (0.1% v/v) and PI (50 µg/ml) and incubated in the dark for 30 minutes. The treated samples were analyzed to assess the cell cycle progression in CytoFlex S (Beckman Coulter) and results were interpreted in CytExpert (Beckman Coulter).

### Caspase 3/7 and Caspase 9 assays

Caspases are a protease family that plays an important role in the process of apoptosis. Caspase kits (Elabscience Biotechnology Inc. Texas, USA) were employed to detect the activity of caspase 3/7 and caspase 9 as per the manufacturer’s instructions.

For the caspase 3/7 assay, A549 cells treated with different concentrations of bixin (400 µM, 800 µM, 1200 µM) were trypsinized and cell pellets were resuspended in cell lysis buffer. The samples were incubated in an ice bath for 30 minutes and centrifuged at 11000 g for 10–15 minutes. The 45 µL reaction buffer and 5 µL of Ac-DEVD-pNA were added to the 50 µL of lysed sample. After an incubation of 1–2 hours at 37 °C, the OD was measured spectrophotometrically at 405 nm in a microplate spectrophotometer (BioTek Epoch, Agilent). For the Caspase 9 assay, the pellets of the treated A549 cells were resuspended in cold lysis buffer and incubated in the ice bath for 30 minutes. The samples were centrifuged at 12000 rpm for 15 minutes. The 50 µL of reaction working solution was added along with 5 µL of Ac-LEHD-pNA. Ac-LEHD-pNA was used as the substrate which was cleaved to produce yellow coloured p-nitroaniline with an absorption maximum at 405 nm recorded in a microplate spectrophotometer (BioTek Epoch, Agilent).

### Gene expression studies by Rt-qPCR

Total RNA was extracted from bixin treated A549 cells using TRIzol reagent (Sigma-Aldrich, Bangalore, Karnataka, India) according to the manufacturer’s instructions. One microgram of extracted RNA was converted into cDNA and Real-time polymerase chain reaction was carried out using PrimeScript RT reagent Kit (Takara, Otsu, Shiga, Japan). The RT-PCR was performed in CFX96 Real Time PCR system (BIO-RAD). The primers designed for the study are shown in Table S1. The 2^−ΔΔCt^ method was used to calculate the relative expression of the BCL-2, BAX, Cyclin D1(CCND1) genes, with GAPDH serving as the reference gene.

### Molecular docking studies

The proteins and ligands for docking studies were retrieved and prepared using *in-silico* tools for efficient interpretation. The protein PDB files for Bcl-2 (PDB ID- 2W3L), Bax (PDB ID-2K7W), and cyclin D1 (PDB ID- 6P8E) were retrieved from RSC Protein DataBank (www.rcsb.org). The retrieved proteins were further prepared using the Pymol Molecular Graphics system (Version 2.0.7). The 3D structure for bixin was downloaded in SDF format from PubChem (https://pubchem.ncbi.nlm.nih.gov/). Docking was performed using Vina Wizard provided in Pyrx^52^ and the interactions of bixin with proteins were visualized in the 3D format using Pymol Molecular Graphics system (Version 2.0.7)^53^ and in 2D format by Discovery Studio Visualizer (Dassault Systèmes, BIOVIA, 2023).

### Molecular dynamics simulations

Molecular dynamics simulations were performed to further validate the interaction between protein-ligand complexes. GROMACS 2020.2 package GROMOS96 43a1 force field was employed to study the interaction of bixin with BAX, BCL-2, and Cyclin D1. After defining the topological parameters using PRODRG server, six MDS runs were performed for each unbound protein and its complex with bixin. The complexes were positioned in a unit cubic box with 1.0 nm from the molecule to the edge. Further, the unit cubic box was solvated with the simple point charge water model. The charge of the above system was neutralized by Cl− ions and energy minimization of 50,000 steps with convergence tolerance of 1000 kJ/mol nm^−1^ was performed. Besides, the system was equilibrated under NVT (Volume, constant particles, and temperature) and NPT (temperature, constant particles, and pressure) for 100 ps. The simulation was carried out for 100 ns and the RMSD, (Root mean square deviation), RMSF (Root mean square fluctuations), Hb (Hydrogen bonds), Rg (Radius of gyration), SASA (Solvent accessible surface areas) were profiled, and visualized in Xmgrace.

### Statistical analysis

The results in each analysis were compared with the control using one-way ANOVA followed by Dunnett’s multiple comparison tests. *p* < 0.05 was considered as significant. All the statistical analyses were performed using the software SPSS Statistics Version 27 provided by IBM. The graphs were visualized in OriginPro provided by OriginLab.

## Conclusion

This research provided for the new insights into the anti-tumor potential of bixin in lung cancer cell lines. It was recognized that bixin exerts significant inhibitory effects on the A549 alveolar adenocarcinoma cell line lowering cell proliferation, migration, and subsequent morphological alterations. Likewise, bixin induced characteristic apoptotic morphological changes including DNA fragmentation, and nuclear condensation in A549. The considerable decrease in mitochondrial membrane potential proved that the bixin is inducing mitochondrial-mediated intrinsic apoptosis. Furthermore, bixin elevated the expression of effector caspases like caspases 3, 7, and 9. Bixin also arrested the cell cycle at the G0/G1 phase and downregulated the expression of CCND1 which encodes cyclin D1, a major cell cycle regulatory protein. The gene expression studies proved that bixin upregulated the expression of proapoptotic BAX , , and downregulated the expression of anti-apoptotic BCL-2.n. The extensive analysis, and gene expression studies coupled with *in-silico* evidenced the apoptotic potential of bixin targeting BAX, BCL-2, and caspases. Furthermore, we also advocate bixin as a Cyclin D1 inhibitor to explore various tumors. This study presents a pioneering effort to discover the antitumor potential of bixin in lung cancer, which needs to be explored comprehensively in the future for its bioavailability and stability, tumor penetration, acute or chronic normal tissue toxicity, off-target effects, tumor heterogeneity, and evolution with emergence of resistant clones.

## Supplementary Information

Supplementary Information.

## Data Availability

All data generated or analysed during this study are included in this published article (and its Supplementary Information files).

## References

[1] Siegel, R. L., Giaquinto, A. N. & Jemal, A. Cancer statistics, 2024. *CA Cancer J. Clin.* **74**, 12–49 (2024). 38230766 10.3322/caac.21820

[2] Global cancer burden growing, amidst mounting need for services. World Health Organization. https://www.who.int/news/item/01-02-2024-global-cancer-burden-growing--amidst-mounting-need-for-services (2024). PMC11115397 38438207

[3] Travis, W. D. Pathology of lung cancer. *Clin. Chest Med.* **23**, 65–81 (2002). 11901921 10.1016/s0272-5231(03)00061-3

[4] Guo, Q. et al. Current treatments for non-small cell lung cancer. *Front. Oncol.* **12**, 945102 (2022). 36033435 10.3389/fonc.2022.945102 PMC9403713

[5] Lee, S. H. Chemotherapy for lung cancer in the era of personalized medicine. *Tuberc. Respir. Dis.* **82**, 179–189 (2019). 10.4046/trd.2018.0068 PMC6609523 30841023

[6] Concato-Lopes, V. M. et al. Trilobolide-6-O-isobutyrate from Sphagneticola trilobata acts by inducing oxidative stress, metabolic changes and apoptosis-like processes by caspase 3/7 activation of human lung cancer cell lines. *Phytomedicine* **128**, 155536 (2024). 38513379 10.1016/j.phymed.2024.155536

[7] Anantharaman, A., Subramanian, B., Chandrasekaran, R., Seenivasan, R. & Siva, R. Colorants and cancer: A review. *Ind. Crops Prod.* **53**, 167–186 (2014).

[8] Varghese, R., Efferth, T. & Ramamoorthy, S. Carotenoids for lung cancer chemoprevention and chemotherapy: Promises and controversies. *Phytomedicine* **116**, 154850 (2023). 37187036 10.1016/j.phymed.2023.154850

[9] Varghese, R. et al. The apocarotenoid production in microbial biofactories: An overview. *J. Biotech.* **10**, 5–16 (2023). 10.1016/j.jbiotec.2023.07.009 37499877

[10] Kapoor, L. & Ramamoorthy, S. Strategies to meet the global demand for natural food colorant bixin: A multidisciplinary approach. *J. Biotech.* **338**, 40–51 (2021). 10.1016/j.jbiotec.2021.07.007 34271054

[11] Kapoor, L. et al. Multispectroscopic, virtual and in vivo insights into the photoaging defense mediated by the natural food colorant bixin. *Food. Funct.* **14**, 319–334 (2023). 36503930 10.1039/d2fo02338e

[12] Kapoor, L. & Ramamoorthy, S. Epidemiological role of plant pigment bixin in adipaging: In vivo pilot study. *Clin. Epidemiol. Glob. Health.* **18**, 101186 (2022).

[13] Li, J. et al. Bixin protects against kidney interstitial fibrosis through promoting STAT6 degradation. *Front. cell dev. biol.* **8**, 576988 (2020). 33313043 10.3389/fcell.2020.576988 PMC7704619

[14] Keita, H. et al. Assessment of the hypoglycemic effect of Bixin in alloxan-induced diabetic rats: in vivo and in silico studies. *J. Biomol. Struct. Dyn.* **39**, 1017–1028 (2021). 32028848 10.1080/07391102.2020.1724567

[15] Pfeffer, C. M. & Singh, A. T. K. Apoptosis: A target for anticancer therapy. *Int. J. Mol. Sci.* **19**, 448 (2018). 29393886 10.3390/ijms19020448 PMC5855670

[16] Gautschi, O., Ratschiller, D., Gugger, M., Betticher, D. C. & Heighway, J. Cyclin D1 in non-small cell lung cancer: A key driver of malignant transformation. *Lung Cancer* **55**, 1–14 (2007). 17070615 10.1016/j.lungcan.2006.09.024

[17] Hengartner, M. O. The biochemistry of apoptosis. *Nature* **407**, 770–776 (2000). 11048727 10.1038/35037710

[18] Cheng, Z. Y. et al. Casticin induces DNA damage and affects DNA repair associated protein expression in human lung cancer A549 cells. *Molecules* **25**, 341 (2020). 31952105 10.3390/molecules25020341 PMC7024307

[19] Kasibhatla, S. et al. Acridine orange/ethidium bromide (AO/EB) staining to detect apoptosis. *CSH Protoc.* **2006**(3), pdp-prot4493 (2006). 10.1101/pdb.prot4493 22485874

[20] Begum, H. M. & Shen, K. Intracellular and microenvironmental regulation of mitochondrial membrane potential in cancer cells. *WIREs Mech. Dis.*10.1002/wsbm.1595r (2023). 36597256 10.1002/wsbm.1595 PMC10176868

[21] Panda, M., Tripathi, S. K. & Biswal, B. K. Plumbagin promotes mitochondrial mediated apoptosis in gefitinib sensitive and resistant A549 lung cancer cell line through enhancing reactive oxygen species generation. *Mol. Bio. Rep.* **47**, 4155–4168 (2020). 32444975 10.1007/s11033-020-05464-w

[22] Luo, M. et al. Antioxidant therapy in cancer: Rationale and progress. *Antioxidants* **11**, 1128 (2022). 35740025 10.3390/antiox11061128 PMC9220137

[23] Tay-Agbozo, S., Street, S. & Kispert, L. The carotenoid Bixin found to exhibit the highest measured carotenoid oxidation potential to date consistent with its practical protective use in cosmetics, drugs and food. *J. Photochem. Photobiol. B Biol.* **186**, 1–8 (2018). 10.1016/j.jphotobiol.2018.06.016 29982093

[24] Balan, D. J. et al. Thymol induces mitochondrial pathway-mediated apoptosis via ROS generation, macromolecular damage and SOD diminution in A549 cells. *Pharm. Rep* **73**, 240–254 (2021). 10.1007/s43440-020-00171-6 33095436

[25] Rieger, A. M., Nelson, K. L., Konowalchuk, J. D. & Barreda, D. R. Modified annexin V/propidium iodide apoptosis assay for accurate assessment of cell death. *J. Vis. Exp.* **50**, 2597 (2011). 10.3791/2597 PMC3169266 21540825

[26] Qu, D. et al. Lappaconitine sulfate induces apoptosis and G0/G1 phase cell cycle arrest by PI3K/AKT signaling pathway in human non-small cell lung cancer A549 cells. *Acta Histochemical* **122**, 151557 (2020). 10.1016/j.acthis.2020.151557 32622431

[27] Chaiputtanapun, P. et al. Biphasic dose-dependent G0/G1 and G2/M cell-cycle arrest by synthetic 2, 3-arylpyridylindole derivatives in A549 lung cancer cells. *Chem. Med. Chem.* **17**, e202200127 (2022). 35595678 10.1002/cmdc.202200127

[28] Pucci, B., Kasten, M. & Giordano, A. Cell cycle and apoptosis. *Neoplasia* **2**, 291–299 (2000). 11005563 10.1038/sj.neo.7900101 PMC1550296

[29] Brentnall, M., Rodriguez-Menocal, L., De Guevara, R. L., Cepero, E. & Boise, L. H. Caspase-9, caspase-3 and caspase-7 have distinct roles during intrinsic apoptosis. *BMC Cell Biol.* **14**, 32 (2013). 23834359 10.1186/1471-2121-14-32 PMC3710246

[30] Parrish, A. B., Freel, C. D. & Kornbluth, S. Cellular mechanisms controlling caspase activation and function. *Cold Spring Harb Perspect Biol.* **5**, a008672 (2013). 23732469 10.1101/cshperspect.a008672 PMC3660825

[31] Radha, G. & Raghavan, S. C. BCL2: A promising cancer therapeutic target. *Biochimica. et Biophys. Acta (BBA)-Rev. Cancer.* **188**, 309–314 (2017). 10.1016/j.bbcan.2017.06.004 28647470

[32] Yamaguchi, R., Lartigue, L. & Perkins, G. Targeting mcl-1 and other BCL-2 family member proteins in cancer therapy. *Pharmacol. Ther.* **195**, 13–20 (2019). 30347215 10.1016/j.pharmthera.2018.10.009

[33] George, J. et al. Comprehensive genomic profiles of small cell lung cancer. *Nature* **524**, 47–53 (2015). 26168399 10.1038/nature14664 PMC4861069

[34] Montalto, F. I. & De Amicis, F. Cyclin D1 in cancer: A molecular connection for cell cycle control, adhesion and invasion in tumor and stroma. *Cells* **9**, 2648 (2020). 33317149 10.3390/cells9122648 PMC7763888

[35] Wen, T., Song, L. & Hua, S. Perspectives and controversies regarding the use of natural products for the treatment of lung cancer. *Cancer Med.* **10**, 2396–2422 (2021). 33650320 10.1002/cam4.3660 PMC7982634

[36] de Araújo, R. S. A. et al. Coumarin derivatives exert anti-lung cancer activity by inhibition of epithelial–mesenchymal transition and migration in A549 cells. *Pharmaceuticals* **15**, 104 (2022). 35056161 10.3390/ph15010104 PMC8782015

[37] Sahayanathan, G. J., Padmanaban, D., Raja, K. & Chinnasamy, A. Anticancer effect of purified polysaccharide from marine clam Donax variabilis on A549 cells. *J. Food Biochem.* **44**, e13486 (2020). 32996209 10.1111/jfbc.13486

[38] Yang, J. et al. Gracillin isolated from Reineckia carnea induces apoptosis of A549 cells via the mitochondrial pathway. *Drug Des Devel Ther.* **15**, 233–243 (2021). 33505158 10.2147/DDDT.S278975 PMC7829125

[39] Ahmad, A. et al. Swertia chirayita suppresses the growth of non-small cell lung cancer A549 cells and concomitantly induces apoptosis via downregulation of JAK1/STAT3 pathway. *Saudi J. Biol. Sci.* **28**, 6279–6288 (2021). 34764752 10.1016/j.sjbs.2021.06.085 PMC8570953

[40] Anantharaman, A., Hemachandran, H., Mohan, S., Ayyathan, D. M. & Siva, R. Induction of apoptosis by apocarotenoids in B16 melanoma cells through ROS-mediated mitochondrial-dependent pathway. *J. Funct. Foods.* **20**, 346–357 (2016).

[41] Zhang, X., Zhao, W. E., Hu, L., Zhao, L. & Huang, J. Carotenoids inhibit proliferation and regulate expression of peroxisome proliferators-activated receptor gamma (PPARγ) in K562 cancer cells. *Arch. Biochem. Biophys.* **512**, 96–106 (2011). 21620794 10.1016/j.abb.2011.05.004

[42] Jan, R. & Chaudhry, G. E. Understanding apoptosis and apoptotic pathways targeted cancer therapeutics. *Adv. Pharm. Bull.* **9**, 205–218 (2019). 31380246 10.15171/apb.2019.024 PMC6664112

[43] Elmore, S. Apoptosis: A review of programmed cell death. *Toxicol. Pathol.* **35**, 95–516 (2007). 10.1080/01926230701320337 PMC2117903 17562483

[44] Czabotar, P., Lessene, G., Strasser, A. & Adams, J. M. Control of apoptosis by the BCL-2 protein family: Implications for physiology and therapy. *Nat Rev Mol Cell Biol* **15**, 49–63 (2014). 24355989 10.1038/nrm3722

[45] Lu, W. L. et al. Cytotoxicity of naringenin induces Bax-mediated mitochondrial apoptosis in human lung adenocarcinoma A549 cells. *Environ. Toxicol.* **35**, 1386–1394 (2020). 32667124 10.1002/tox.23003 PMC7689782

[46] Yang, S. et al. Sesamin induces A549 cell mitophagy and mitochondrial apoptosis via a reactive oxygen species-mediated reduction in mitochondrial membrane potential. *Korean J. Physiol. Pharmacol.* **24**, 223–232 (2020). 32392913 10.4196/kjpp.2020.24.3.223 PMC7193912

[47] Gao, X., Leone, G. W. & Wang, H. Cyclin D-CDK4/6 functions in cancer. *Adv. Cancer Res.* **148**, 147–169 (2020). 32723562 10.1016/bs.acr.2020.02.002

[48] Liao, K., Li, J. & Wang, Z. Dihydroartemisinin inhibits cell proliferation via AKT/GSK3β/cyclinD1 pathway and induces apoptosis in A549 lung cancer cells. *Int. J. Clin. Exp. Pathol.* **7**, 684 (2014). PMC4314032 25674233

[49] Guo, M. et al. Cediranib induces apoptosis, G1 phase cell cycle arrest, and autophagy in non-small-cell lung cancer cell A549 in vitro. *Biomed. Int. Res.* **2021**, 5582648 (2021). 10.1155/2021/5582648 PMC8024085 33860036

[50] Shanmugapriya, K., Kim, H. & Kang, H. W. In vitro antitumor potential of astaxanthin nanoemulsion against cancer cells via mitochondrial mediated apoptosis. *Int. J. Pharm.* **560**, 334–346 (2019). 30797074 10.1016/j.ijpharm.2019.02.015

[51] Fang, J. et al. Acid ground nano-realgar processed product inhibits breast cancer by inducing mitophagy via the p53/BNIP3/NIX pathway. *J. Cell Mol. Med.* **27**, 3478–3490 (2023). 37610095 10.1111/jcmm.17917 PMC10660646

[52] Dallakyan, S. & Olson, A. J. Small-Molecule Library Screening by Docking with PyRx. *Methods Mol. Biol.* **1263**, 243–250 (2015) 25618350 10.1007/978-1-4939-2269-7_19

[53] Schrödinger, L., & DeLano, W. PyMOL. (2020). http://www.pymol.org/pymol

