# Supplementary material for: Deciphering the effects of bixin on pulmonary alveolar adenocarcinoma migration and proliferation via targeting BAX/BCL-2 and Cyclin D1
Source: Sci Rep. 2025 Apr 29;15:15109. doi: 10.1038/s41598-025-96788-9 (PMC12041254; doi:10.1038/s41598-025-96788-9)
Supplement: Supplementary file 1 — Supplementary Information. [file 41598_2025_96788_MOESM1_ESM.docx]

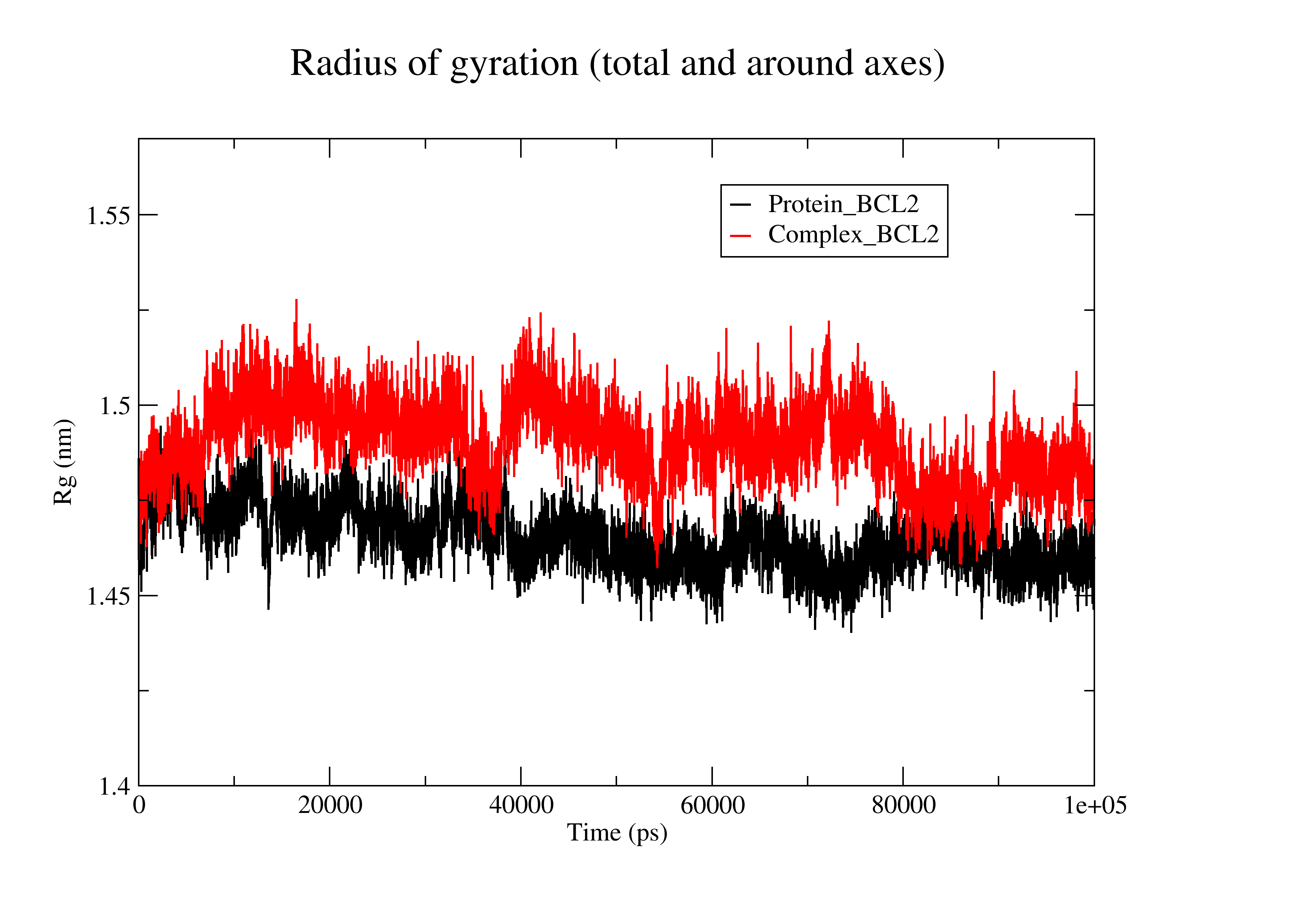

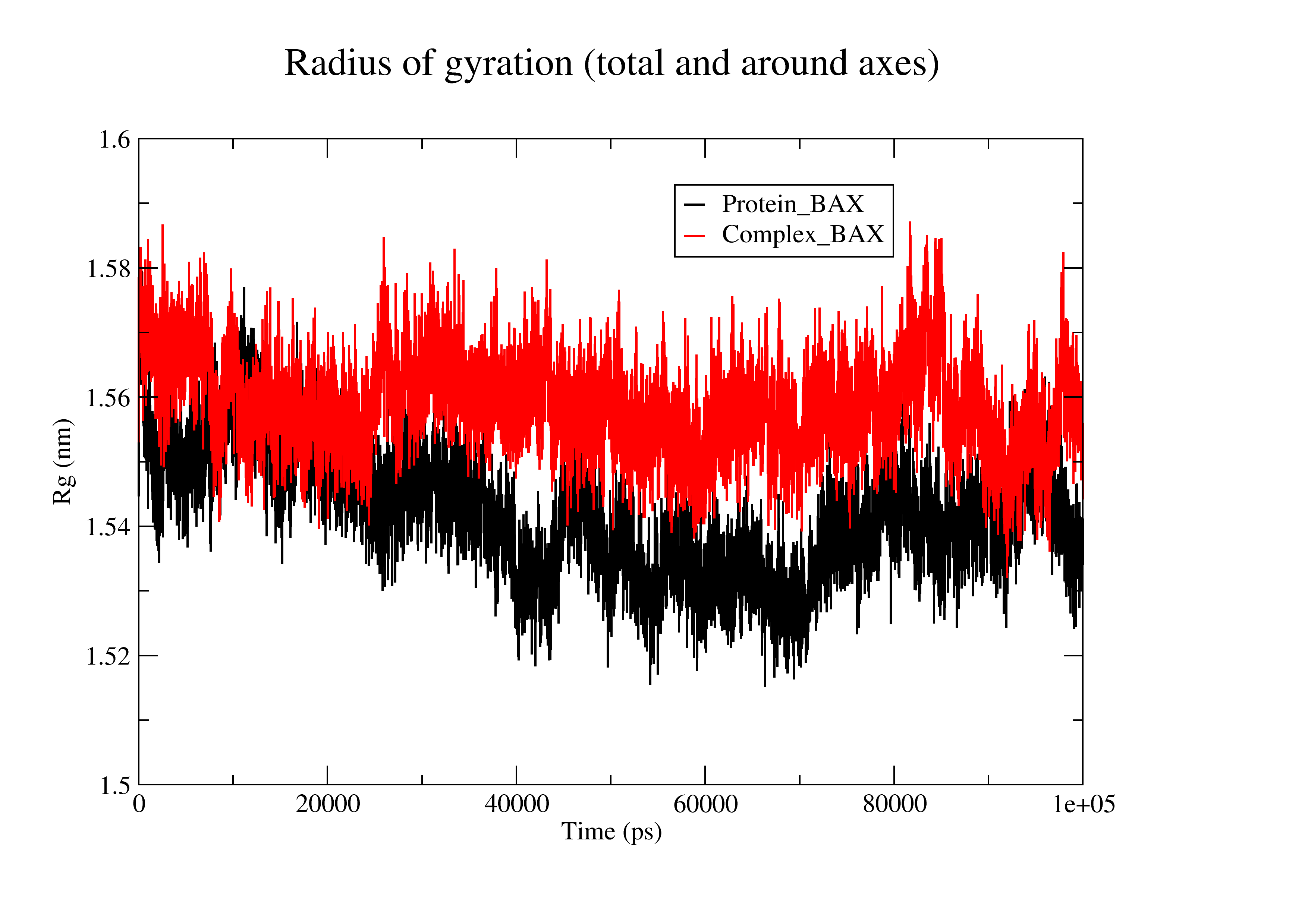

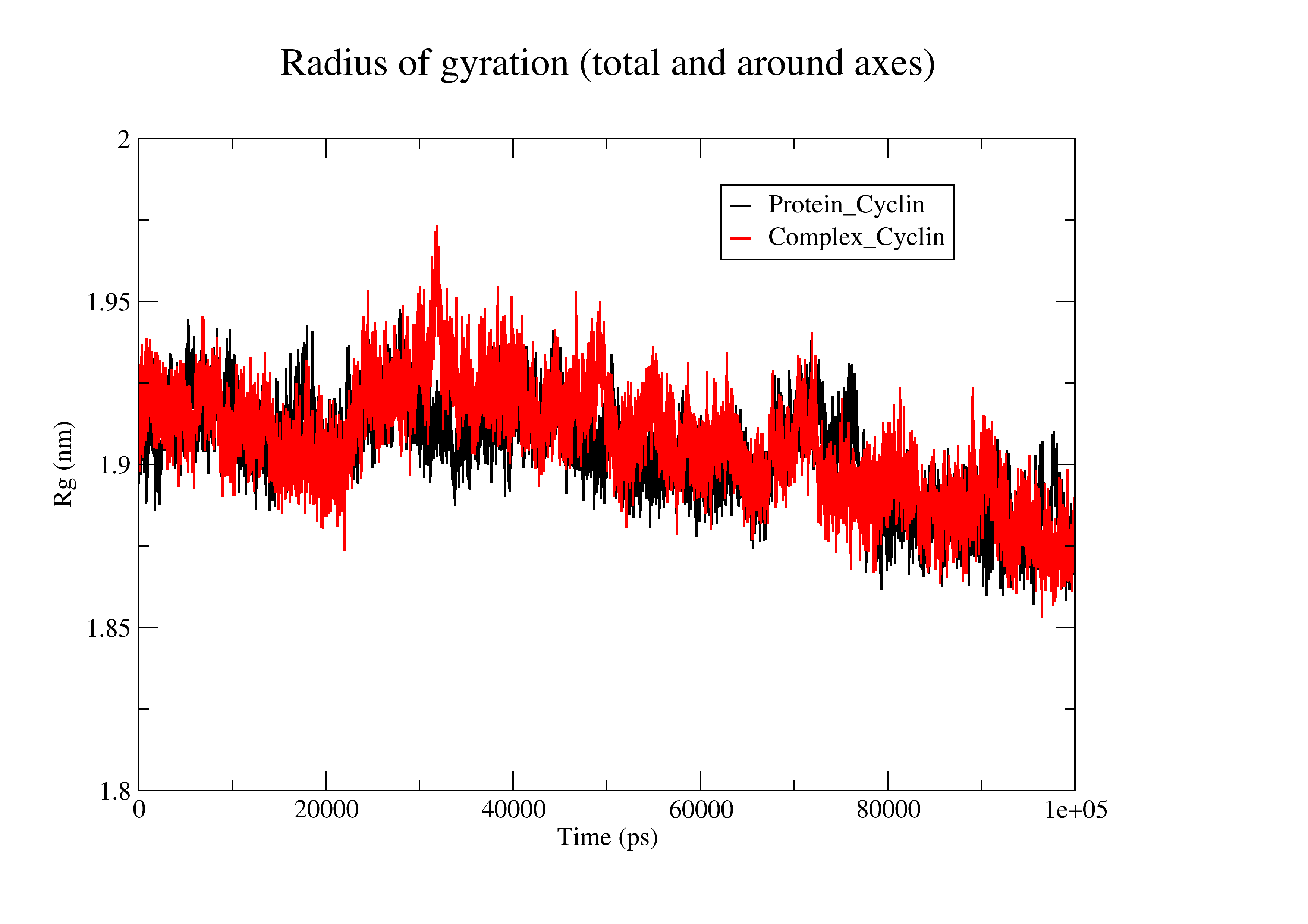


b,

a,


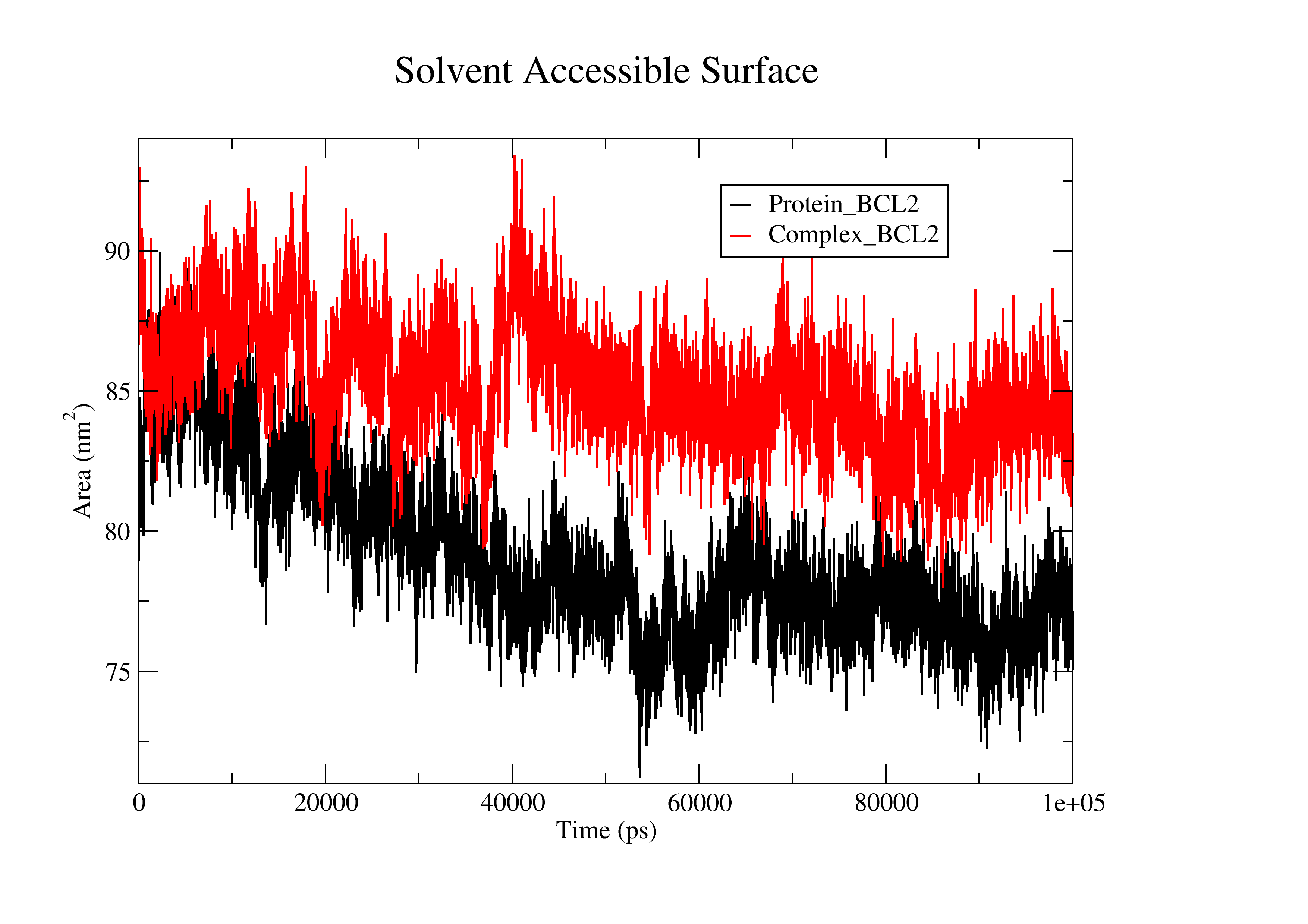

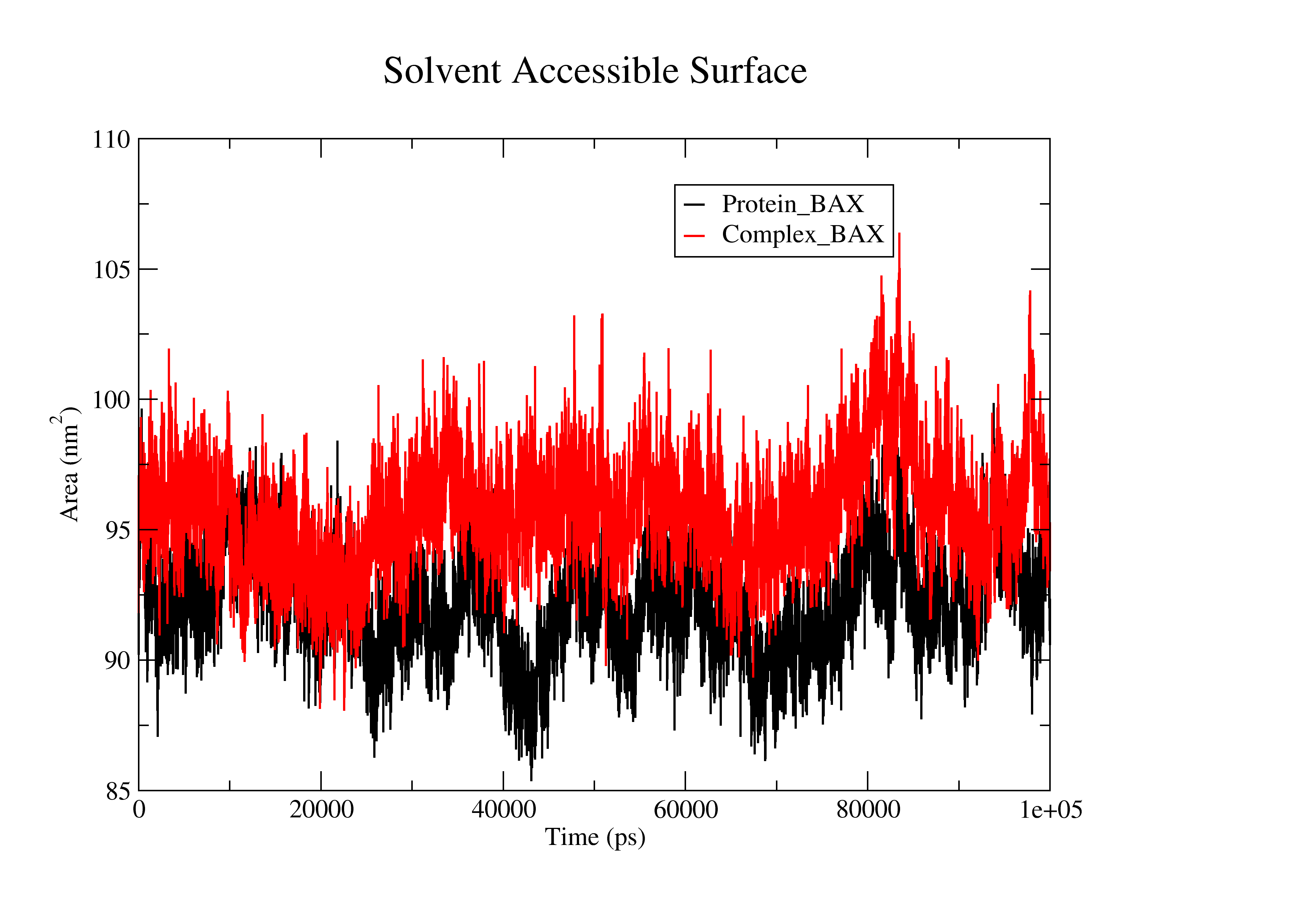

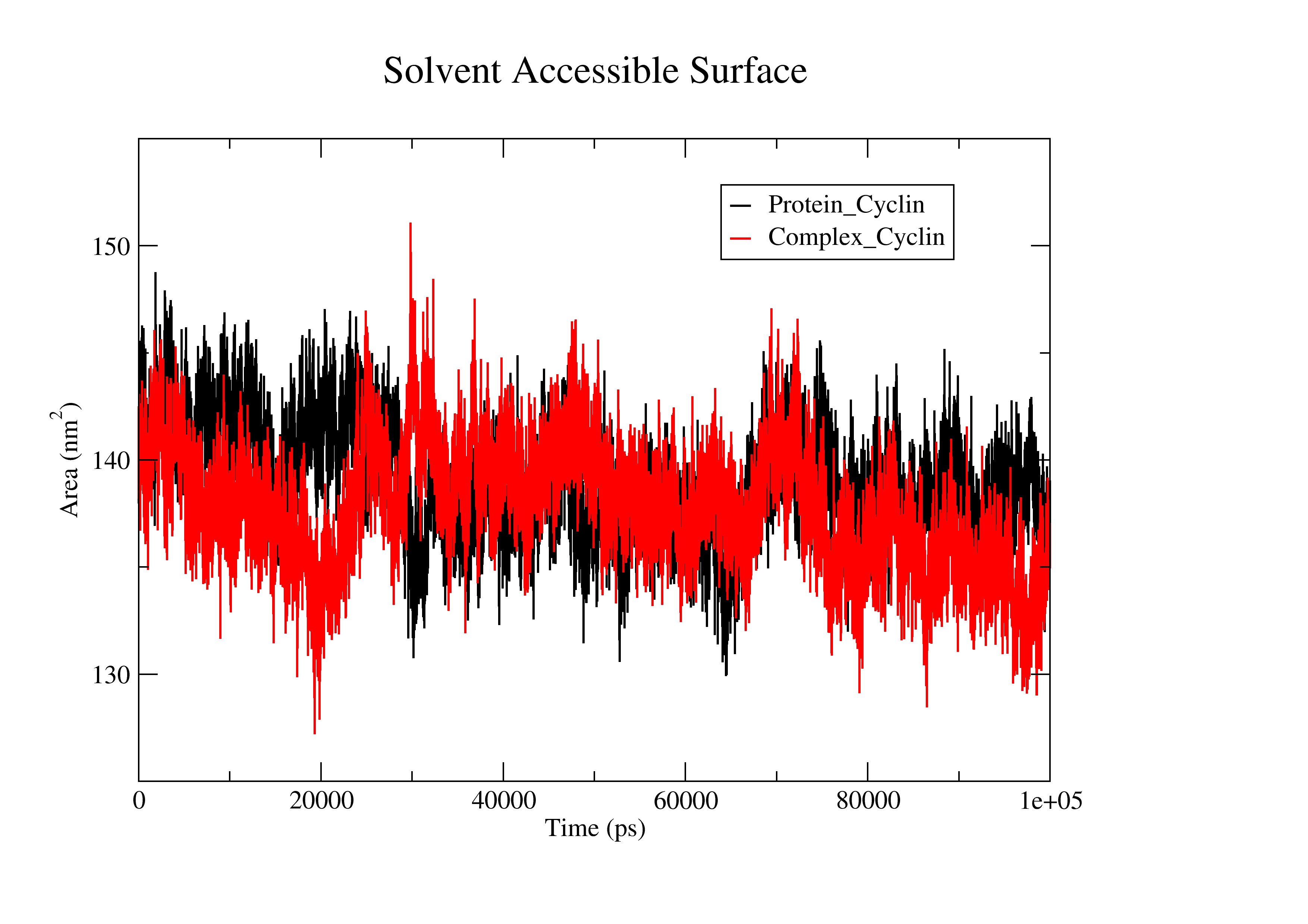


c,


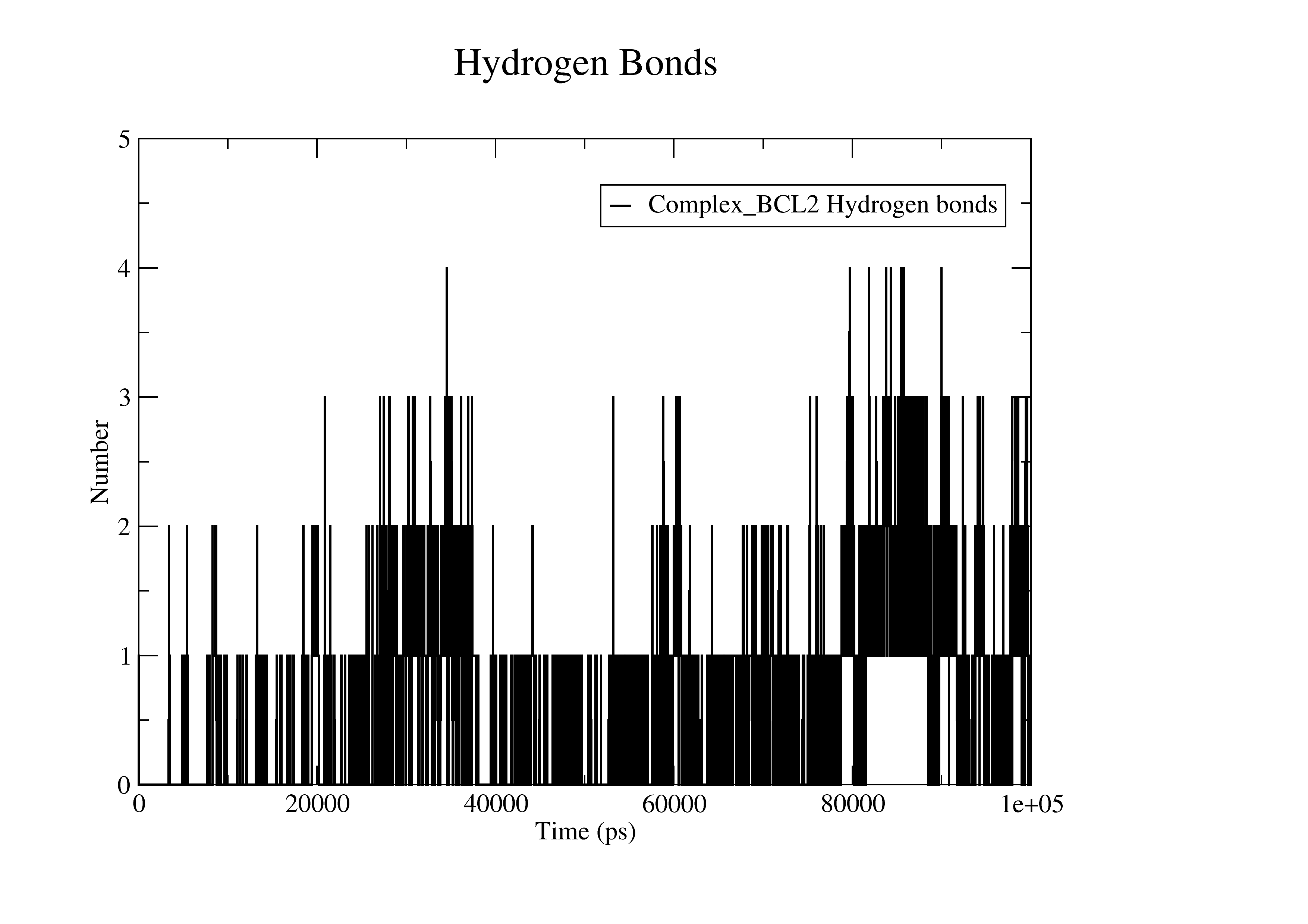

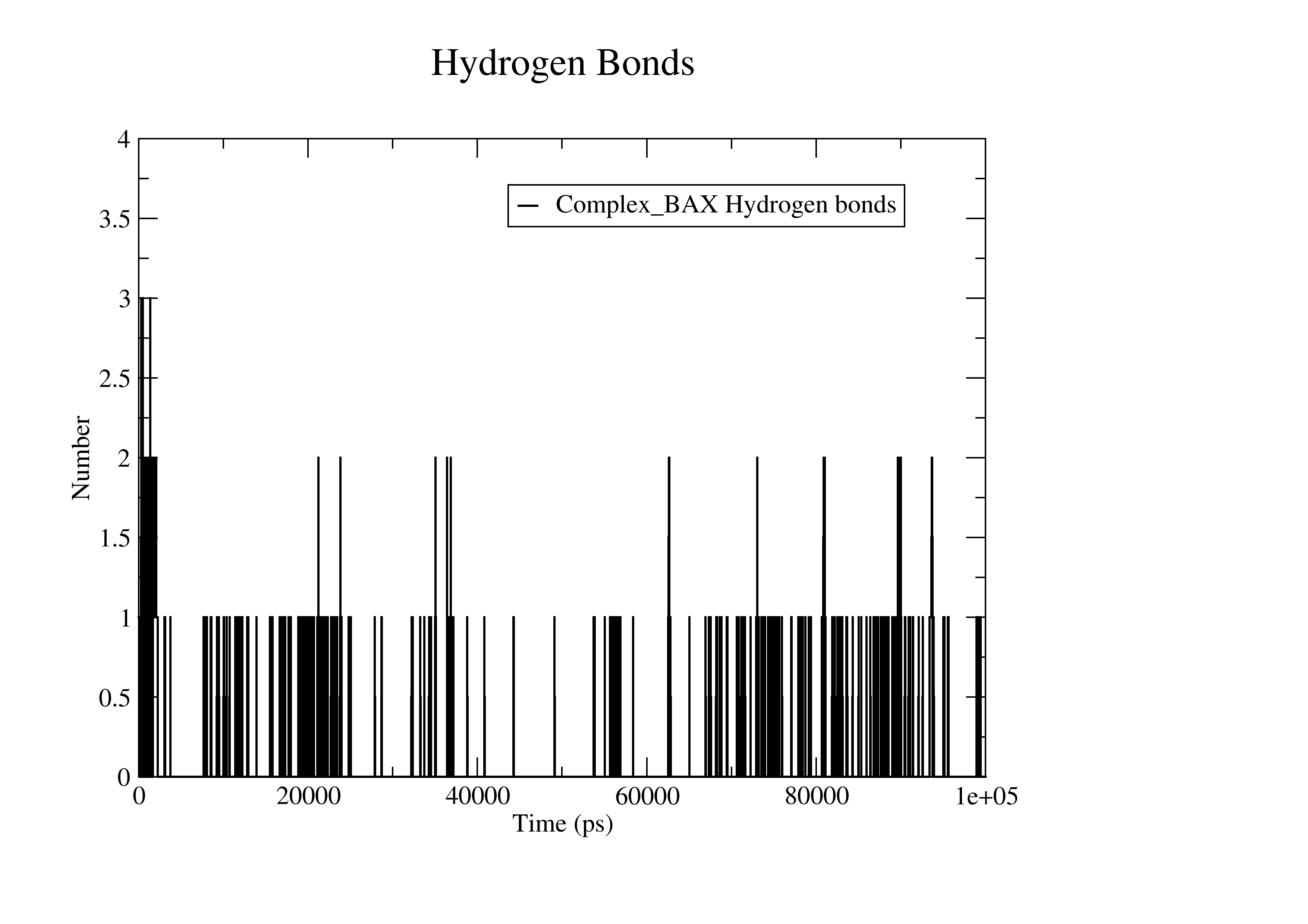

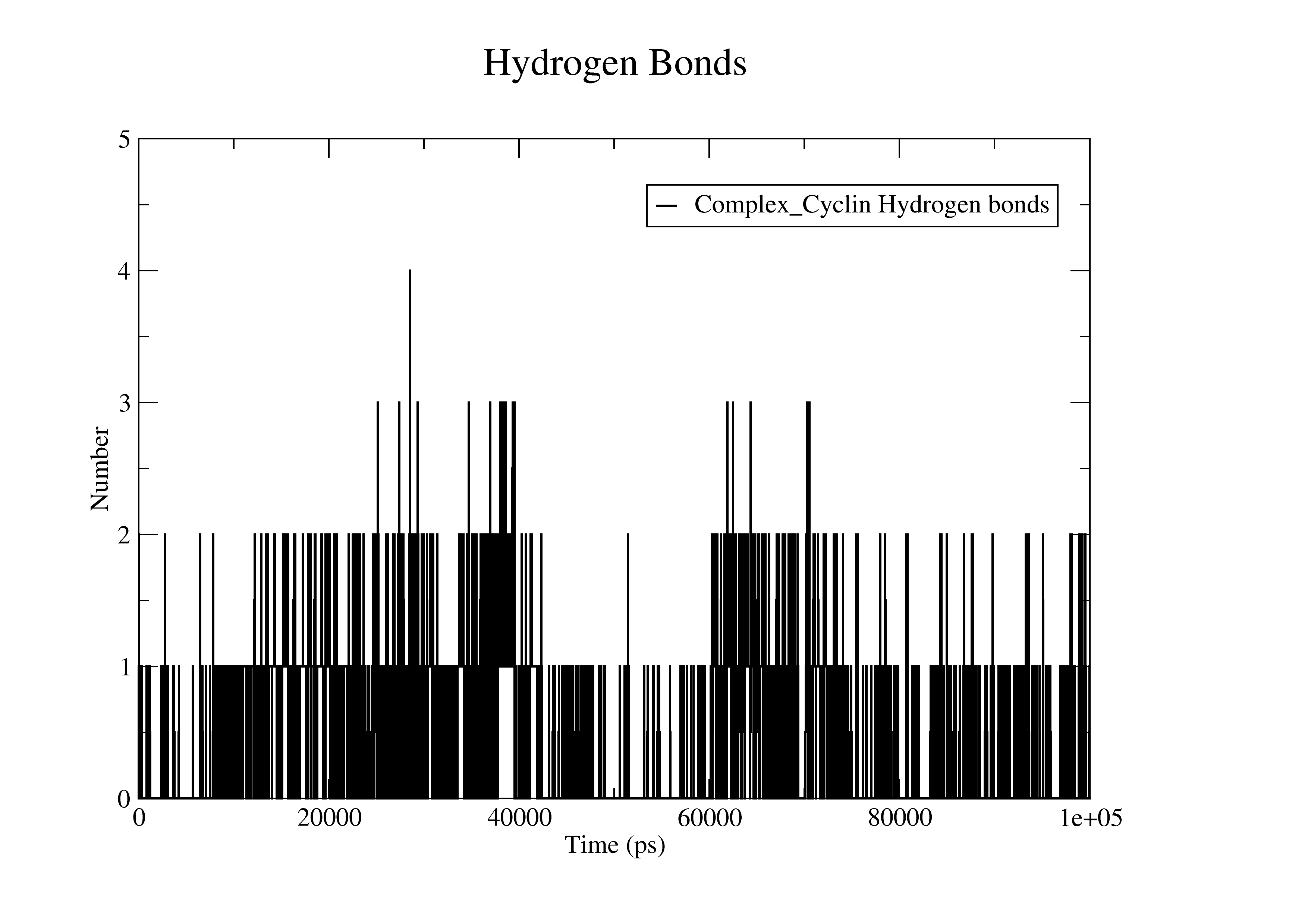


**Supplementary Fig. S1.** The MDS analysis of the interaction of bixin with BCL2, BAX and cyclin D1. a, Rg profiling of apo protein and complex b, SASA of apo protein and complex c, Hydrogen bonds in the complex during the interaction.

| Gene | Primer | Sequence |
| --- | --- | --- |
| BCL-2 | Forward | CTTTTGCTGTGGGGTTTTGT |
| BCL-2 | Reverse | GTCATTCTGGCCTCTCTTGC |
| BAX | Forward | GGAGCTGCAGAGGATGATTG |
| BAX | Reverse | CCTCCCAGAAAAATGCCATA |
| CCND1(Cyclin D1) | Forward | GCGAGGAACAGAAGTGC |
| CCND1 (Cyclin D1) | Reverse | GAGTTGTCGGTGTAGATGC |
| GAPDH | Forward | GAAGGTGAAGGTCGGAGT |
| GAPDH | Reverse | GAAGATGGTGATGGGATTTC |

**Supplementary Table S1**. Primers used for real-time quantitative PCR analysis.
